# Immobilization of a [Co^III^Co^II^(H_2_O)W_11_O_39_]^7–^ Polyoxoanion for the Photocatalytic Oxygen Evolution Reaction

**DOI:** 10.1021/acsmaterialsau.2c00025

**Published:** 2022-05-25

**Authors:** Sreejith P. Nandan, Nadiia I. Gumerova, Jasmin S. Schubert, Hikaru Saito, Annette Rompel, Alexey Cherevan, Dominik Eder

**Affiliations:** † Institute of Materials Chemistry, TU Wien, Getreidemarkt 9/BC/02, 1060 Vienna, Austria; ‡ Universität Wien, Fakultät für Chemie, Institut für Biophysikalische Chemie, Althanstraße 14, 1090 Vienna, Austria; § Institute for Materials Chemistry and Engineering, Kyushu University, 6-1 Kasugakoen, Kasuga, Fukuoka 816-8580, Japan

**Keywords:** heterogeneous photocatalysis, homogeneous photocatalysis, polyoxometalate, molecular metal oxide, water oxidation catalysis, co-catalyst, cluster, APTES, surface modification

## Abstract

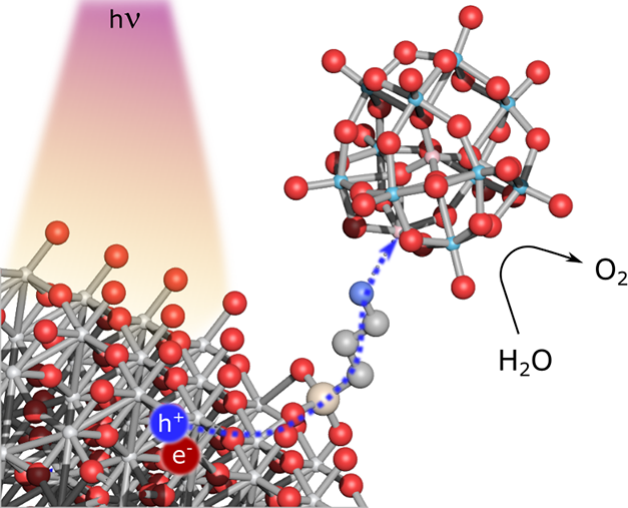

The ongoing transition to renewable energy sources and the implementation of artificial photosynthetic setups call for an efficient and stable water oxidation catalyst (WOC). Here, we heterogenize a molecular all-inorganic [Co^III^Co^II^(H_2_O)W_11_O_39_]^7–^ ({Co^III^Co^II^W_11_}) Keggin-type polyoxometalate (POM) onto a model TiO_2_ surface, employing a 3-aminopropyltriethoxysilane (APTES) linker to form a novel heterogeneous photosystem for light-driven water oxidation. The {Co^III^Co^II^W_11_}-APTES-TiO_2_ hybrid is characterized using a set of spectroscopic and microscopic techniques to reveal the POM integrity and dispersion to elucidate the POM/APTES and APTES/TiO_2_ binding modes as well as to visualize the attachment of individual clusters. We conduct photocatalytic studies under heterogeneous and homogeneous conditions and show that {Co^III^Co^II^W_11_}-APTES-TiO_2_ performs as an active light-driven WOC, wherein {Co^III^Co^II^W_11_} acts as a stable co-catalyst for water oxidation. In contrast to the homogeneous WOC performance of this POM, the heterogenized photosystem yields a constant WOC rate for at least 10 h without any apparent deactivation, demonstrating that TiO_2_ not only stabilizes the POM but also acts as a photosensitizer. Complementary studies using photoluminescence (PL) emission spectroscopy elucidate the charge transfer mechanism and enhanced WOC activity. The {Co^III^Co^II^W_11_}-APTES-TiO_2_ photocatalyst serves as a prime example of a hybrid homogeneous–heterogeneous photosystem that combines the advantages of solid-state absorbers and well-defined molecular co-catalysts, which will be of interest to both scientific communities and applications in photoelectrocatalysis and CO_2_ reduction.

## Introduction

Owing to the ever-growing demand for renewable energy sources, the research community has been exploring processes that enable the generation of solar fuels. Among various solar-powered means to produce H_2_, photocatalytic water splitting stands out as the most direct and cost-effective approach.^1−3^ This light-driven, energetically uphill reaction involves two redox processes: water oxidation to form O_2_—termed as the oxygen evolution reaction (OER)—and H^+^ reduction to yield H_2_—termed as the hydrogen evolution reaction (HER). Due to the sluggish kinetics of water oxidation and the necessity of four-electron transfer, it is the OER that is often considered to be the bottleneck of water splitting.^4^ Even though OER does not generate a fuel, it provides reducing equivalents required for its production, e.g., by the reduction of protons to H_2_ in water splitting or by reduction of CO_2_ to CO or other carbonaceous products in artificial photosynthesis. Given the importance and challenging nature of this reaction, a bulk of research works have been devoted to the search for an efficient light-driven water oxidation catalyst (WOC).

Among various families of photocatalysts, metal oxides such as SrTiO_3_, NaTaO_3_, WO_3_, and BiVO_4_ are considered forerunners for heterogeneous WOC due to their suitable electronic structures, earth abundance, and redox stability.^5^ However, even though many reported photocatalysts can act as efficient light-absorbers, their performance is often limited by the low efficiencies of the WOC step, i.e., the activation of water molecules at the solid/liquid interface and their step-wise oxidation.^6^ To overcome this issue, co-catalysts are often employed in the form of small nanoparticles attached to the support surface.^7^ Not only do they provide better-suited active and adsorption sites, they also facilitate the extraction of photoexcited charge carriers to the reaction centers. The purposeful design of active and selective co-catalysts is, however, often complicated by their poorly defined surface structures, which ultimately limits the degree of control over their performance. The implementation of structurally defined molecular co-catalysts emerges as a promising alternative approach to tackle this challenge.^65^

In parallel to the development of heterogeneous photosystems, various transition metal-based complexes have been demonstrated to act as efficient and quick (photo)catalysts for water oxidation under strictly homogeneous conditions.^8−10^ However, the WOC performance of these molecular species is often compromised by the instability of the organic ligands toward oxidation by the reactive intermediates formed upon illumination.^11^ In view of this limitation, polyoxometalates (POMs) have more recently emerged as promising WOC candidates as they combine high structural tunability and superior redox stability^12^ owing to their rigid metal-oxo frameworks. After the first all-inorganic WOC-active [{Ru_4_O_4_(OH)_2_(H_2_O)_4_}(γ-SiW_10_O_36_)_2_]^10–^ cluster had been reported in 2008,^13^ the first earth-abundant Co-based [Co_4_(H_2_O)_2_(PW_9_O_34_)_2_]^10–^ has triggered extensive interest in Co-containing POM-WOCs.^14^ To date, many other Co-based POMs have been reported for homogeneous (light-driven) water oxidation, and their overall performance was investigated with regard to POM’s structural type,^15^ accessibility of Co centers,^16^ and pH values.^17^

Despite these developments, two issues still hinder a broader implementation of POM-WOCs: self-aggregation of POM clusters under the turnover conditions and the poor stability of molecular photosensitizers.^18^ Both aspects are highly detrimental and often lead to rapid deactivation of the photosystems, which limit their long-term performance. In order to address these challenges and combine the advantages of heterogeneous and homogeneous approaches to photocatalysis, a recent perspective discussed the prospects of POM heterogenization on the surface of photoactive supports.^19^ In this setup, POM takes the role of a structurally and compositionally well-defined co-catalyst, while the semiconducting support complements by providing an efficient and stable light absorption. As an added benefit, immobilization is expected to improve the structural stability of individual POM clusters and further allow for control over the electronic communication between the absorber and the catalyst.

We recently summarized previous examples of POMs composited within various solid-state matrices.^19^ Several polyoxotungstate and polyoxomolybdate clusters have been successfully deposited onto metal oxides,^20−27^ carbon nitrides,^28,29^ metal–organic, and covalent–organic frameworks;^30−34^ however, mostly (photo)electrocatalytic, sensing, and solar cell applications have been targeted so far. Also, most of the protocols relied on physisorption/electrostatic binding,^35,36^ which can be detrimental to the stability of the resulting photosystems. In this work, we have covalently attached a WOC-active POM onto photoactive metal oxide surfaces using a linker molecule. We evaluated the resulting composite toward light-driven water oxidation, for which the role of photosensitizer is taken by the inorganic support. We investigated the synergistic properties and the charge transfer dynamics between the two components as well as the stabilization effect upon attachment.

As the first example of this approach in photocatalytic WOC, we chose the well-studied TiO_2_ (anatase) substrate as it has an appropriate electronic structure for water splitting^37^ and is a very efficient, nontoxic photocatalyst with a large surface area and excellent redox stability.^38^ To provide chemically tunable binding sites for the POM anions, 3-aminopropyltriethoxysilane (APTES) was chosen as the linker given its bifunctional nature: the ethoxide moiety can covalently attach to the hydroxylated oxide surface upon condensation of the ethoxy groups,^39,40^ while the amino moiety on the opposite end provides strong Lewis base sites for the POM anchoring. As for the choice of POM cluster, several criteria needed to be considered: the availability of complementary binding sites (i.e., Lewis acid centers) along with a labile ligand (such as H_2_O) for the APTES attachment, established WOC performance under homogeneous conditions, and high negative charge of the anion to allow for hydrolytic stability under neutral-to-basic pH of the WOC reaction. Considering these, we chose K_7_[Co^III^Co^II^(H_2_O)W_11_O_39_], which has a monosubstituted Keggin anion featuring a Co(III) central ion and a [Co^II^(H_2_O)]^2+^ unit that replaces one of the twelve peripheral [W^VI^=O]^4+^ addenda ion groups.^17^ In addition, this polyanion has a high negative charge of −7, and with its two Co heteroions in two different oxidation states (+II and +III), it has been reported to be the most promising among Co-containing POMs for WOC applications,^17^ which makes it an excellent choice for our heterogenization approach.

## Experimental Section

### Chemical Reagents

All the precursor materials used for the synthesis of K_7_[Co^III^Co^II^(H_2_O)W_11_O_39_]·14H_2_O ({Co^III^Co^II^W_11_}) and {Co^III^Co^II^W_11_}-APTES-TiO_2_ were obtained from commercial suppliers. Anatase TiO_2_, Na_2_WO_4_·2H_2_O, Co(OAc)_2_·4H_2_O, K_2_S_2_O_8_, KNO_3_, [Ru(bpy)_3_]Cl_2_·6H_2_O, and Na_2_S_2_O_8_ were all of highest purity and purchased from Merck (Sigma). Hexane (HPLC-pure) used for precipitation was purchased from VWR.

### Synthesis Protocols

#### Synthesis of K_7_[Co^III^Co^II^(H_2_O)W_11_O_39_]·14H_2_O

The synthesis was done using a modified protocol based on Baker and McCutcheon.^41^ For this, 19.8 g (0.06 mol) Na_2_WO_4_·2H_2_O was dissolved in 40 mL of H_2_O, and then by the addition of glacial acetic acid, the solution pH was adjusted between 6.5 and 7.5 and heated to near-boiling. Afterward, 2.5 g (0.01 mol) of Co(OAc)_2_·4H_2_O dissolved in 13 mL of warm H_2_O was added dropwise into the tungstate solution while stirring. A pink precipitate got formed and redissolved quickly, forming a dark green solution. The mixture was then heated to reflux for 10 min, and insoluble material was filtered out. Seven grams (0.026 mol) of K_2_S_2_O_8_ was added^17^ while heating the solution at 80 °C and then kept until boiling. Once the reaction solution changed its color from green to dark brown, the boiling was continued for another 5 min, which was followed by filtration. The filtrate was then kept for heating again until boiling. Lastly, 25 mL of hot saturated KNO_3_ was added, and the mixture was cooled, leading to the formation of a brown precipitate. This dark brown solid was then filtered out. Later, this solid was added to water and heated to 90 °C, stirred for a few minutes, and filtered, and the filtrate was collected. The filtrate was then kept for cooling, and the precipitated solid was removed by filtration. The obtained clear brown solution was kept for crystallization by evaporation at room temperature, and dark brown cubic single crystals were formed. The unit cell parameters calculated from single crystal XRD are *a* = *b* = *c* = 21.55 Å, α = γ = β = 90° and are consistent with those previously reported for {Co^III^Co^II^W_11_} (CCDC code 915800).^17^ Elemental analysis found (calculated) in %: K: 8.6 (8.2); Co: 3.7 (3.5); W: 60.2 (60.8).

#### Functionalization of TiO_2_ with APTES

The synthesis was done using a modified protocol based on Kockmann et al.^42^ For this, 500 mg of anatase nanoparticles was taken in 50 mL of absolute ethanol in a round bottom (RB) flask and kept for sonication for 1 h. After sonication, an excess (1.5 mL) of APTES (considering 1 mol APTES per mol TiO_2_) was taken and added to the reaction medium. The RB was refluxed overnight at 85 °C, and after cooling the RB, 50 mL of hexane was added to the suspension to induce precipitation. After a few minutes, it was centrifuged at 6500 rpm for 15 min at room temperature (RT), and the residue was collected. This residue was washed using absolute ethanol and was centrifuged two more times using hexane. Finally, the precipitate was collected and kept for drying overnight in a vacuum oven at room temperature.

#### Immobilization of {Co^III^Co^II^W_11_} onto APTES-TiO_2_

Following the protocol by Yang et al.,^43^ 250 mg of APTES-TiO_2_ was dispersed in an aqueous {Co^III^Co^II^W_11_} solution (125 mg of {Co^III^Co^II^W_11_} and 25 mL of water). The pH was monitored throughout and kept in the slightly acidic range (between 6.3 and 6.7) by adding 0.1 M HCl, making sure that the POM maintains its integrity without getting hydrolyzed. The solution was stirred for 24 h at RT, followed by centrifuging, washing with 100 mL of water, and drying overnight to yield the {Co^III^Co^II^W_11_}-APTES-TiO_2_ composite.

### Photocatalytic Water Oxidation

For conducting photocatalytic water oxidation reaction experiments, the reaction solutions were prepared in a closed, two-necked glass reactor with an outer water-cooling (15 °C) jacket. This 2 mL reaction media was then deaerated using Ar purging (100 mL/min) for 30 min until it reached almost-zero O_2_ concentration. The reaction setup was kept under darkness for 20 min to get a constant baseline before illumination was started to trigger a photocatalytic reaction. The evolved O_2_ was recorded using a O_2_ sensor (FireStingO2, Pyroscience) inserted to the reaction medium (i.e., volume above the solution) through a viton septum placed in a screw cap on one of the necks of the reactor. It works on the principle of fluorescence quenching of an indicator at the tip of the sensor, which depends on the concentration of O_2_ surrounding it. The advantages include sensor staying inside the reactor, which can have in situ detection of O_2_ amounts, with a 1 s data collection interval. The preparation of reaction solutions for both homogeneous and heterogeneous reactions is given as follows. For homogeneous WOC, the reaction medium was prepared by taking 20 μM (details in the SI) concentrations of POM catalysts dissolved in 80 mM aqueous borate buffer solution (pH 8), containing 1 mM Ru[bpy]_3_Cl_2_ (bpy = 2,2′-bipyridine) as the photosensitizer (PS) and 5 mM Na_2_S_2_O_8_ as a sacrificial agent (SA). The illumination was done using a monochromatic LED light source (445 ± 13 nm, power = 64 mW/cm^2^, incident light intensity = 129 mW, Thorlabs SOLIS). For heterogeneous WOC, 1 mg of the POM-immobilized TiO_2_ sample was dispersed in 2 mL of 10 mM Na_2_S_2_O_8_ aqueous solution. The illumination was done using a monochromatic LED light source (365 ± 6 nm, power = 183 mW/cm^2^, incident light intensity = 366 mW, Thorlabs SOLIS).

### Photoluminescence Measurements

A photocatalytic WOC reaction was conducted with {Co^III^Co^II^W_11_}-APTES-TiO_2_ in 10 mM Na_2_S_2_O_8_ reaction solution diluted with 3 × 10^–3^ M (in 0.01 M NaOH) terephthalic acid (TA) solution. Once the UV illumination starts and the charge carriers are photogenerated, TA can get converted to 2-hydroxyterephthalic acid (TA-OH) if ·OH are generated. As TA-OH is fluorescent, PL emission can be used to quantify the amount of generated ·OH. Similarly, the protocol was followed for TiO_2_ and 10 mM Na_2_S_2_O_8_ (blank) solution as well. After illuminating the OER reaction solutions (with TA) for 30 min, it was centrifuged at 5600 rpm for 20 min, and the supernatant was then syringe-filtered (0.45 μm pore) to remove any suspended particles. The PL emission of this solution was probed with an excitation wavelength of 315 nm.

## Results and Discussion

### Synthesis, Structure, and Characterization of K_7_[Co^III^Co^II^(H_2_O)W_11_O_39_]

**Figure 1:**
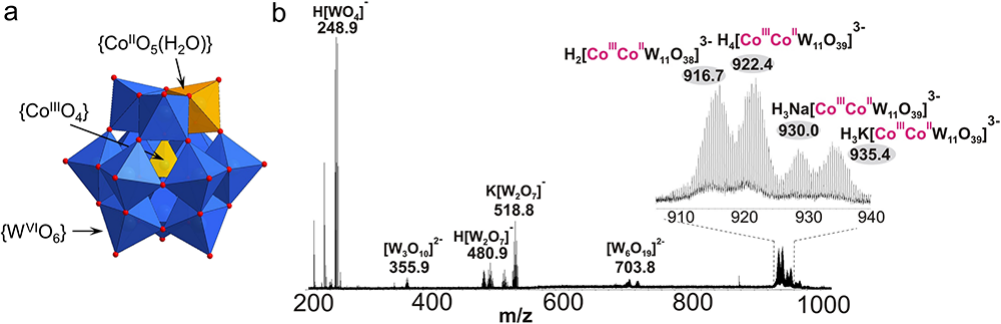
[Co^III^Co^II^(H_2_O)W_11_O_39_]^7–^ polyanion: (a) polyhedral model of the polyanion, demonstrating the Keggin structure with peripheral W=O^4+^ replaced by a [Co^II^(H_2_O)]^2+^ heteroion. Labels: red, O; blue, {WO_6_}; yellow (peripheral), {Co^II^O_5_(H_2_O)}; yellow (central), {Co^III^O_4_}; hydrogen atoms are omitted for clarity. (b) ESI-MS spectrum of {Co^III^Co^II^W_11_} in the region 200–1000 *m*/*z* recorded in the negative mode in water at pH ≈ 6, exhibiting peak envelopes of Keggin anions at *m*/*z* = 922.4, 930.0, and 935.4.

The chosen [Co^III^Co^II^(H_2_O)W_11_O_39_]^7–^ polyanion has a monosubstituted Keggin structure with 11 {W^VI^O_6_} and one {Co^II^O_5_(H_2_O)} octahedra surrounding the central {Co^III^O_4_} tetrahedron as schematically shown in Figure 1a. The structure of the cluster was confirmed using single-crystal X-ray diffraction (XRD) and attenuated total reflectance Fourier-transform IR spectroscopy (ATR-FTIR), and was further characterized by X-ray photoelectron spectroscopy (XPS) and powder XRD (details in the SI and Figure S1).^17^ Elemental constituents derived from inductively coupled plasma mass spectrometry (ICP-MS) and total reflection X-ray fluorescence spectroscopy (TXRF, Table S2) further confirm its composition and the presence of two Co centers. The stability of {Co^III^Co^II^W_11_} in water at pH ≈ 6 relevant to WOC studies was investigated by electrospray–ionization mass spectrometry (ESI-MS, Figure 1b). The ESI-MS spectrum recorded in negative mode exhibits a series of the peaks’ envelopes at *m*/*z* between 910 and 940, which can be unambiguously assigned to the triply charged anions H_4-*x*_K(Na)_*x*_[Co^III^Co^II^W_11_O_39_]^3–^ (*x* = 0, 1) with experimental (calculated) *m*/*z* values of 922.4 (922.4), 930.0 (930.0), and 935.4 (935.4), substantiating the presence of an intact POM cluster. Thermogravimetric analysis (TGA) was used to elucidate the number of crystal waters, annotating the overall formula of the POM to be K_7_[Co^III^Co^II^(H_2_O)W_11_O_39_]·14H_2_O (Figure S1d and Table S1).

### APTES Functionalization on the TiO_2_ Surface

**Figure 2:**
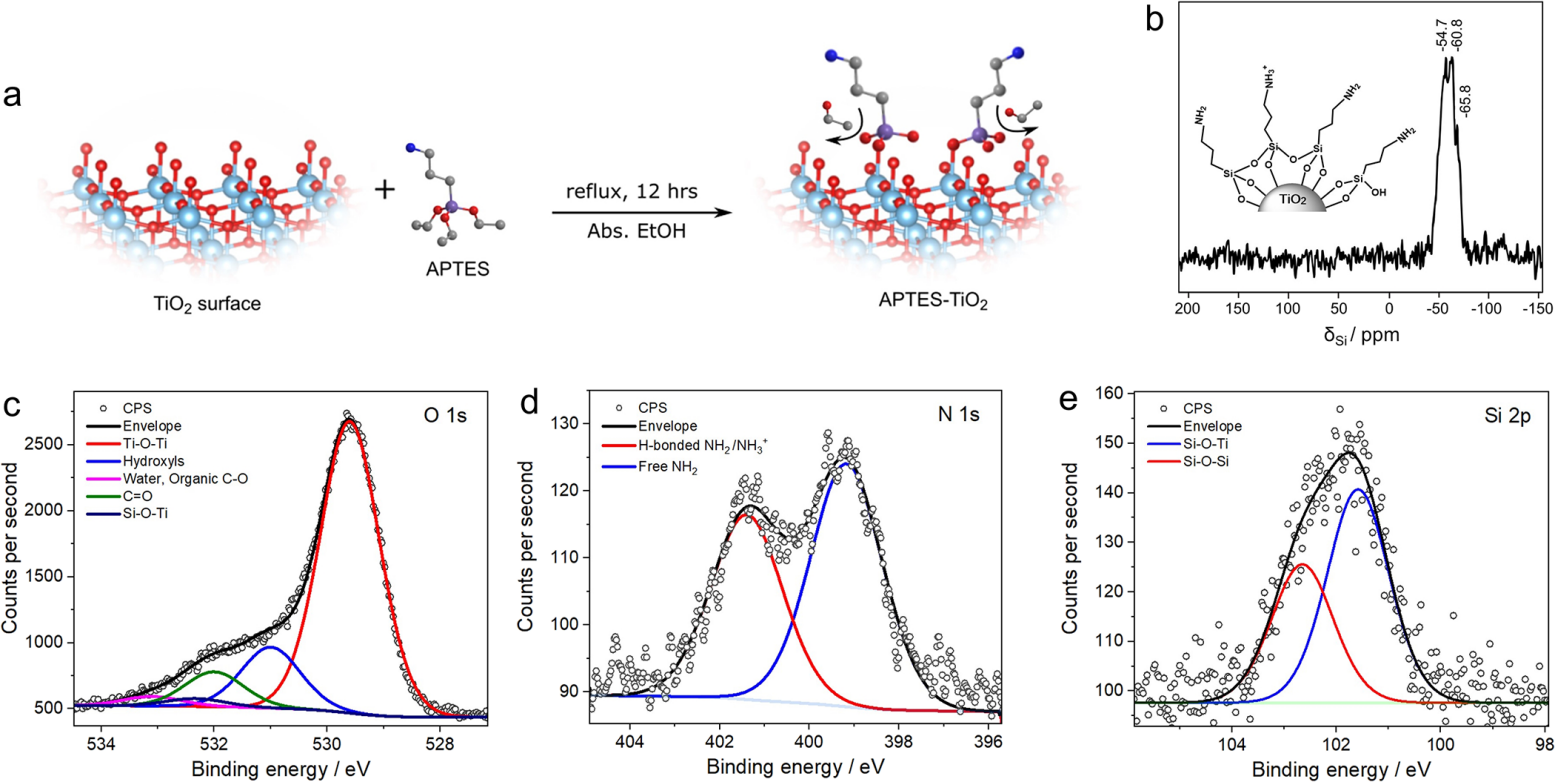
Schematic and characterization of APTES attachment onto TiO_2_:(a) Illustration of APTES functionalization onto TiO_2_ surface; labels: light blue, Ti; red, O; gray, C; dark blue, N; purple, Si. (b) ^29^Si solid-state NMR of APTES-TiO_2_ showing a broad peak centered around −60 ppm, corresponding to bi-/tridentate attachment of APTES onto TiO_2_; the inset schematic suggests the bonding at the interface (based on the solid-state ^29^Si NMR), and the state of the terminal amino groups (based on N 1s XPS). (c) O 1s XPS spectrum indicating the presence of Si–O–Ti and Ti–O–Ti linkages present in APTES-TiO_2_; the presence of adventitious organic contamination is denoted as C–O and C=O contributions. (d) N 1s XPS spectrum showing the presence of both free NH_2_ and H-bonded NH_2_, or NH_3_^+^ and (e) Si 2p XPS spectrum confirming the presence of Si(IV).

An APTES monolayer was deposited onto the TiO_2_ support via ethoxy group hydrolysis and condensation to surface hydroxyls^39,44^ to ensure specific and selective attachment of the POM clusters (Figure 2a and the Experimental Section). Various characterization techniques including ^29^Si solid-state nuclear magnetic resonance (NMR) spectroscopy, XPS, and ICP-MS confirmed the APTES coverage. The ^29^Si NMR spectrum of APTES-TiO_2_ in Figure 2b shows only one broad peak, with a chemical shift around −60 ppm, which is consistent with the presence of APTES.^40^ Based on the ^29^Si chemical shifts measured for unattached APTES (−37 ppm) as well as those attached in the monodentate (−43 ppm), bidentate (−59 ppm), or tridentate (−67 ppm) modes,^45^ the chemical shifts (−54.7, −60.8, and –65.8 ppm) strongly suggest bidentate/tridentate binding of APTES onto TiO_2_ surface.

XPS was performed to gain further insight into the binding nature at the APTES/TiO_2_ interface. The survey spectrum (Figure S2) shows signals for Si 2p, N 1s, Ti 2p, and O 1s, indicating the presence of both composite components. The detailed O 1s spectrum (Figure 2c) shows a broad peak at 529.5 eV, which arises from TiO_2_ bulk,^46,47^ and a broad shoulder peak at 532.0 eV that belongs to the TiO_2_–surface hydroxides, organic species from the adventitious carbon (C–O), and moisture.^48^ The O 1s profile further contains a contribution at 532.3 eV, which can be assigned to Si–O–Ti^39,49^ in line with the NMR data. The N 1s peak (Figure 2d) contains two pronounced peaks at 399.6 and 401.3 eV, indicating two different chemical environments: the first peak being the characteristic of free NH_2_,^39,50^ while the latter can be assigned to H-bonded or protonated amino groups,^50−53^ which may coexist after the attachment due to the close proximity of the APTES molecules. The Si 2p spectrum (Figure 2e) shows a peak centered around 102 eV, which can be assigned to the Si–O–Ti bonds,^39,50^ indicating that most Si atoms are grafted on TiO_2_. A broad peak shape (full-width half maximum of ca. 2.1 eV), however, points toward the existence of two overlapping contributions, in which the shoulder at 102.8 eV being indicative of Si–O–Si bonds formed between closely packed APTES moieties (Figure 2b, inset). The overall data thus show that APTES attaches onto TiO_2_ as a monolayer via Si ions (covalent Si–O–Ti bonds), while amino moieties stay available for POM anchoring (Figure 2a).

### Immobilization of {Co^III^Co^II^W_11_} onto APTES-TiO_2_

**Figure 3:**
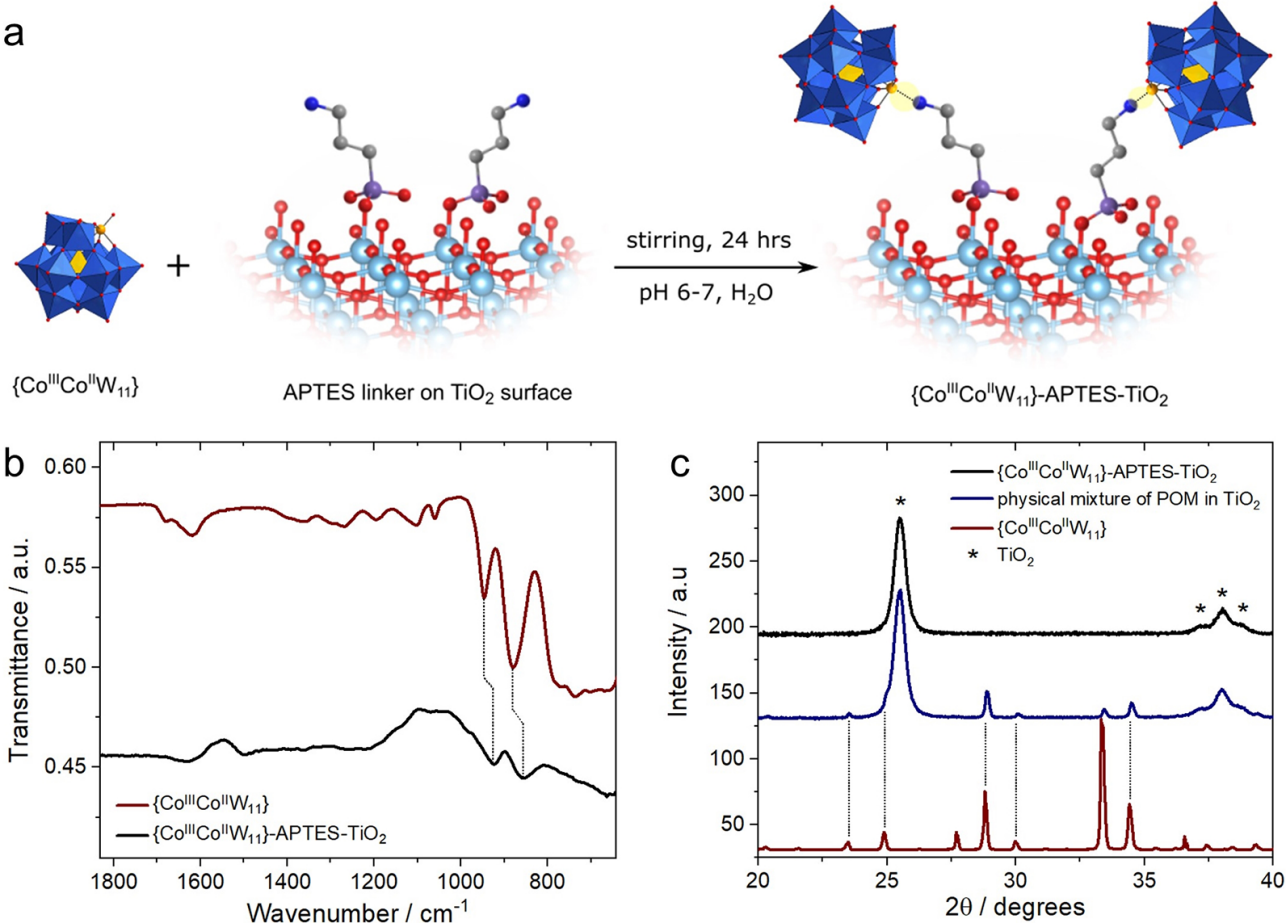
Schematic of attachment and characterizations of {Co^III^Co^II^W_11_}-APTES-TiO_2_: (a) Schematic representation of the {Co^III^Co^II^W_11_} attachment onto APTES-TiO_2_, highlighting the new coordinate bond formed between NH_2_ (of APTES) and Co(II) of the POM via the aqua ligand replacement. Labels: light blue, Ti; red, O; gray, C; dark blue, N; purple, Si; yellow, Co^II^; blue octahedra, {WO_6_}; yellow tetrahedron, {Co^III^O_4_}. (b) ATR-FTIR spectra of the {Co^III^Co^II^W_11_}-APTES-TiO_2_ composite subtracted by APTES-TiO_2_ IR spectrum and that of {Co^III^Co^II^W_11_} POM. (c) Powder XRD of {Co^III^Co^II^W_11_}-APTES-TiO_2_, compared with a physical mixture of 10 wt % of {Co^III^Co^II^W_11_} and APTES-TiO_2_.

Once the TiO_2_ surface was functionalized by APTES linkers, we proceeded with {Co^III^Co^II^W_11_} immobilization (refer to the Experimental Section). In the [Co^III^Co^II^(H_2_O)W_11_O_39_]^7–^ cluster, due to the weak coordination bond between peripheral Co(II) ions and the H_2_O ligand, the aqua ligand can be replaced by a stronger NH_2_ from APTES, resulting in a H_2_N: → Co(II) dative bond,^54−56^ which we confirmed preliminarily by reacting {Co^III^Co^II^W_11_} with a model *n*-butylamine (Figure S3).^57^ With this choice of the components and attachment procedure, the formation of the {Co^III^Co^II^W_11_}-APTES-TiO_2_ composite is driven by self-assembly (Figure 3a). No such coordination is possible for the parent [PW_12_O_40_]^3–^ clusters as W(VI) has no free d-orbitals and its terminal O ions are connected by a very stable double bond (details in the SI).

Figure 3b shows the ATR-FTIR spectrum of the {Co^III^Co^II^W_11_}-APTES-TiO_2_ composite, from which the spectrum of APTES-TiO_2_ is subtracted. The difference spectrum indicates the presence of bands in the 850–950 cm^–1^ regime, which corresponds to W=O and W–O–W vibrations, suggesting the presence of an intact {Co^III^Co^II^W_11_} cluster in the composite. The slight shift in peak positions indicates minor structural rearrangements associated with the loss of crystal water during immobilization, which is expected from the presence of the individual POM clusters on the surface. To confirm the molecular nature of immobilization and to examine if any POM aggregation takes place, powder XRD and scanning transmission electron microscopy (STEM) were utilized. Figure 3c shows the diffraction pattern of the physical mixture prepared by grinding 10 wt % {Co^III^Co^II^W_11_} in TiO_2_ as a reference, which contains the typical peaks of both anatase and {Co^III^Co^II^W_11_}. Note that the POM-related peaks are absent in the {Co^III^Co^II^W_11_}-APTES-TiO_2_ composite despite a relatively high loading of 14 wt % {Co^III^Co^II^W_11_} (see the ICP-MS discussion below). This strongly suggests that {Co^III^Co^II^W_11_} clusters are present in their molecular form. Elemental energy-dispersive X-ray spectroscopy (EDS) mapping (Figure S4) shows uniform and matching spatial distribution of Co, W, and Ti on the nanoscale, which further confirms a highly homogeneous coverage and dispersion of POM clusters over the titania matrix.

**Figure 4:**
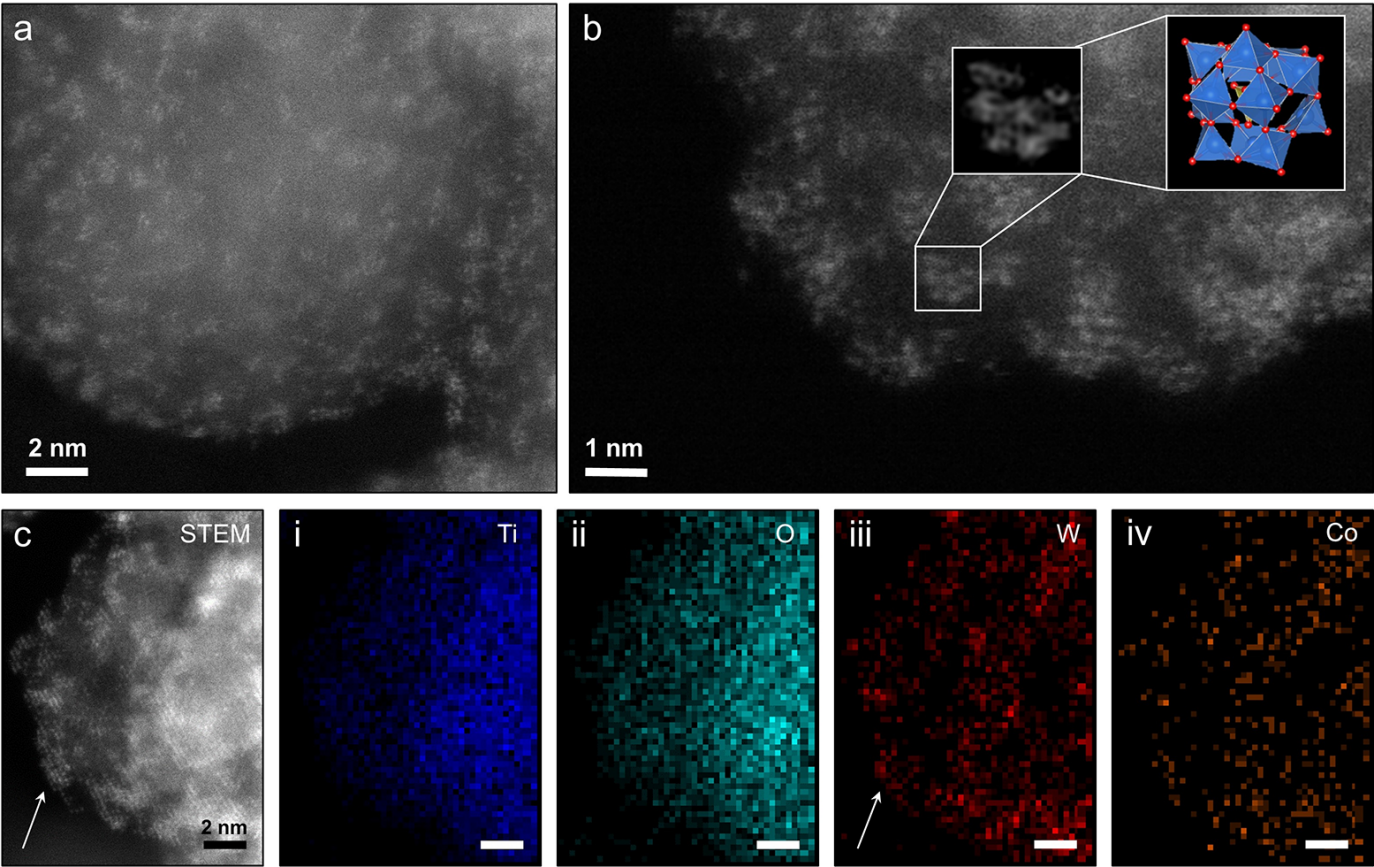
Visualization of {Co^III^Co^II^W_11_} on the surface: (a) High-resolution HAADF-STEM image of {Co^III^Co^II^W_11_}-APTES-TiO_2_ composite, showing collections of multiatomic clusters decorating the TiO_2_ surface. (b) A magnified region of a TiO_2_ nanoparticle’s edge resolving {Co^III^Co^II^W_11_} anions; the squared area and its Fourier filtered image allow reconstruction of the orientation of an individual {Co^III^Co^II^W_11_} cluster corresponding to the configuration of the eight W ions distinguishable in STEM; the three remaining W ions of the POM structure are hidden behind the eight that are visible, and the Co atoms cannot be resolved due to their low atomic number (27) similar to that of Ti (22). (c) STEM-EDS data featuring elemental maps of (i) Ti, (ii) O, (iii) W, and (iv) Co; areas of high W concentration correspond well to the surface-attached POM clusters observable in STEM mode (see arrow) due to the high *Z*-contrast between Ti (22) and W (74).

To derive an atomistic picture of the surface-anchored POM clusters, we employed aberration-corrected high-angle annular dark-field scanning transmission electron microscopy (HAADF-STEM; details in the SI). A high-resolution micrograph of the {Co^III^Co^II^W_11_}-APTES-TiO_2_ sample in Figure 4a reveals multiple assemblies of bright spots distributed evenly over the surface of the supporting TiO_2_ nanoparticle (more images in Figure S5). Considering the strong *Z*-contrast difference between W and Ti along with the fact that the approximate lateral size of each assembly is around 1 nm, these formations likely correspond to individual POM clusters decorating the surface, while each of the spots – to a single W ion. Figure 4b shows a close-up micrograph and confirms the presence of multiple POM anions attached to the surface in various orientations, in line with the rough and uneven surface of the TiO_2_ support. The Fourier-filtered image of the inset area overlaid with a structural model of a {Co^III^Co^II^W_11_} cluster demonstrates an excellent match, which further corroborates the successful heterogenization and intact structure of the POMs after attachment. A series of STEM-EDS maps acquired with atomic resolution (Figure 4c) verify the chemical composition of the clusters and confirm the presence of Co centers (additional elemental maps in Figure S6).

The amount of {Co^III^Co^II^W_11_} on the TiO_2_ surface was quantified by ICP-MS and TXRF (Table 1). Both methods result in an average loading of 14 wt % (details in the SI). Moreover, a ratio of 1:7.4 for POM to APTES can be calculated, suggesting that on average, seven NH_2_ groups are available to accommodate each of the {Co^III^Co^II^W_11_} anions. Next, we estimated the theoretical maximum loading for {Co^III^Co^II^W_11_} on TiO_2_, considering a monolayer adsorption model (details in the SI), by taking 1.9 nm^2^ as the footprint of a Keggin-structured POM^58^ and 78.63 m^2^/g as the surface area of TiO_2_ measured according to Brunauer–Emmett–Teller (BET) theory.^59^ This yields a theoretical value of 20.8 wt %, which—considering additional repulsion between the close-packed POM anions at high loadings—can be seen as an upper loading limit. The experimentally obtained value of 14 wt % agrees well with a dense monolayer of POM clusters on TiO_2_ and is in line with the homogeneous distribution of the clusters revealed by the STEM and EDS in Figure 4.

**Table 1:** Amount of {Co^III^Co^II^W_11_} Loaded (wt %) onto TiO_2_ Calculated from ICP-MS and TXRF Measurements

	loading^a^ of {Co^III^Co^II^W_11_} on APTES-TiO_2_ (wt %)	
technique used	Co^b^	W^b^
ICP-MS	12.9	14.1
TXRF	11.2	14.5

a Loading of *x* wt % implies that *x* mg of {Co^III^Co^II^W_11_} is attached to 100 mg of APTES-TiO_2_.

b Calculation done based on Co/Ti and W/Ti experimental signal values. The loading values based on W/Ti ratios are more reliable considering the much higher mass and atomic ratio of W to that of Co in {Co^III^Co^II^W_11_}.

**Figure 5:**
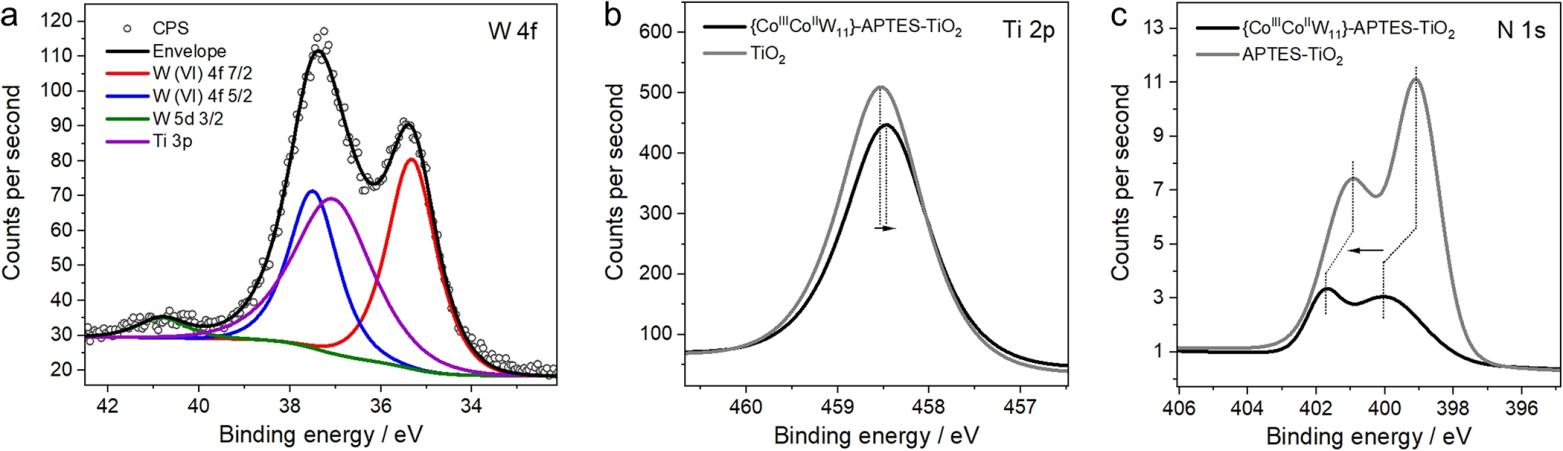
XPS spectra of {Co^III^Co^II^W_11_}-APTES-TiO_2_: (a) W 4f XPS spectrum showing the presence of W(VI) and Ti(IV), (b) Ti 2p XPS spectrum of {Co^III^Co^II^W_11_}-APTES-TiO_2_ compared with TiO_2_ showing a slight shift in peak positions, and (c) N 1s XPS spectrum of {Co^III^Co^II^W_11_}-APTES-TiO_2_ compared with that of APTES-TiO_2_.

The binding modes between POM and APTES were investigated by XPS. Figure 5a shows the W 4f spectrum of {Co^III^Co^II^W_11_}-APTES-TiO_2_ with the characteristic peaks for W 4f 5/2 and W 4f 7/2 at 37.4 and 35.4 eV, respectively. Note that these peaks are slightly redshifted in comparison to the bare POM (Figure S7b), which indicates an electronic communication between the clusters and the support. Moreover, the peak intensity ratio of W 4f 5/2 to W 4f 7/2 is considerably higher for the composite (1.2) compared to that of the bare POM (0.75^60^). This can be explained by an overlapping contribution of the Ti 3p peak of the TiO_2_ substrate (Figure S7b). Note that we can also exclude the presence of WO_3_, which shows peaks at 38.5 and 36.1 eV.^60^Figure 5b shows the Ti 2p spectrum of the composite, which only contains a characteristic Ti(IV) signal; however, we observe a slight shift of the peak maximum to lower binding energies compared to bare TiO_2_. This shift is indicative of surface modification caused by the presence of electron-rich Ti ions and is in line with the formation of Ti–O–Si bonds at the APTES-TiO_2_ surface.^39^ It can also be observed that the Ti peak shows a small decrease in intensity, which could be related to the existence of the surface-attached APTES and {Co^III^Co^II^W_11_} layers that limit the mean free path of the photoelectrons.^50^ While a reliable interpretation of Co 2p spectra is complicated by the low intensity of the signal (Figure S7e), the N 1s spectra of APTES-TiO_2_ and {Co^III^Co^II^W_11_}-APTES-TiO_2_ provide useful insights (Figure 5c). The N 1s peak at lower binding energy is clearly diminished in the composite compared to APTES-TiO_2_, which implies a decrease in the number of free NH_2_ upon cluster loading, in line with the proposed attachment mode via H_2_N: → Co(II) bonds. The N 1s spectrum of the composite also shows a shift in peak positions to higher binding energies, which can be indicative of a transfer of electron density from N to Co. Overall, XPS results are in accordance with the schematic in Figure 3a and confirm the immobilization of {Co^III^Co^II^W_11_} via NH_2_ groups of APTES as well as corroborate the attachment of APTES onto TiO_2_ surface via covalent Si–O–Ti bonds.

### Photocatalytic WOC Activity

**Figure 6:**
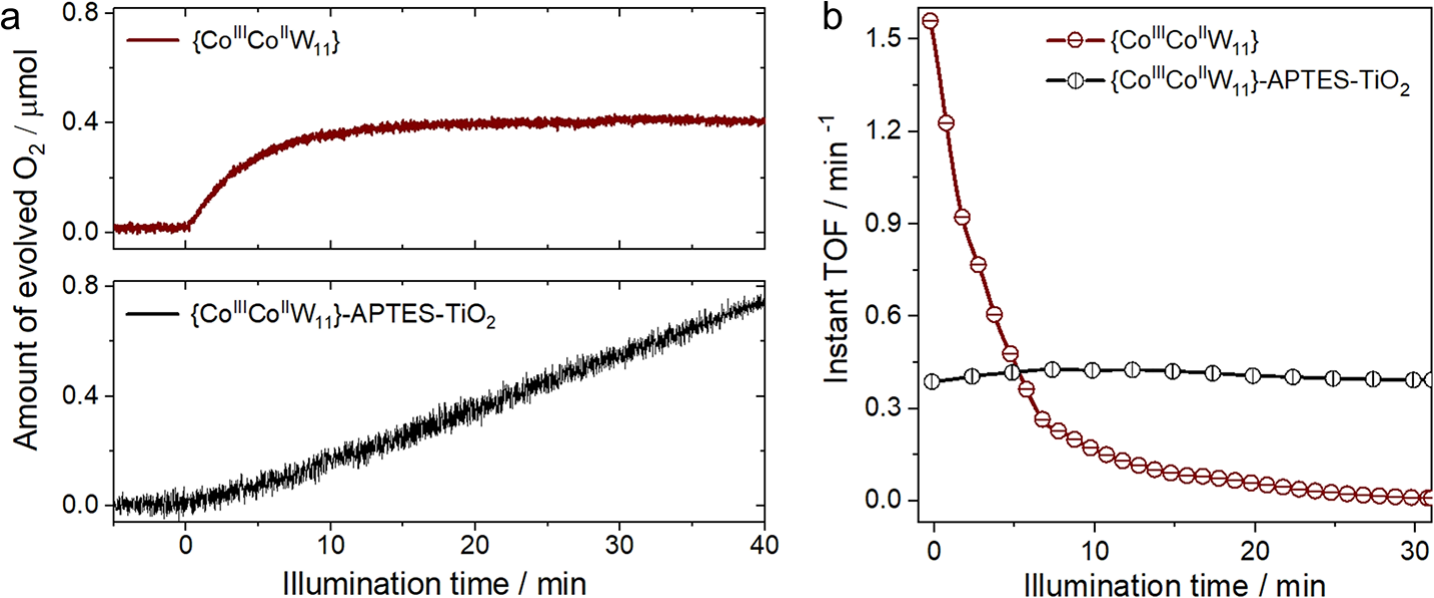
WOC activity of {Co^III^Co^II^W_11_} under both homogeneous and heterogeneous conditions: (a) Top: Homogeneous WOC activity of 20 μM {Co^III^Co^II^W_11_} under 445 nm visible light illumination; bottom: Heterogeneous WOC activity of the {Co^III^Co^II^W_11_}-APTES-TiO_2_ composite under 365 nm UV light illumination; details in the Experimental Section. (b) Instant TOF vs illumination time (min) plotted for both homogeneous and heterogeneous WOC reactions.

First, we verified visible light-driven WOC activity of the chosen POM cluster under homogeneous conditions using 20 μM {Co^III^Co^II^W_11_} in pH 8 borate (80 mM) buffer solution, 1 mM [Ru(bpy)_3_]Cl_2_ as a molecular photosensitizer responsible for the light absorption, and 5 mM Na_2_S_2_O_8_ as a sacrificial oxidant (details in the Experimental Section). Figure 6a (top) shows the resulting O_2_ evolution profile: after initial high-performance WOC within the first minutes of illumination, the photosystem deactivates completely after ca. 30 min with the amount of generated O_2_ saturating at around 0.4 μmol. This performance can be translated into an O_2_ evolution turnover number (TON) of 9.8 and an instant turnover frequency (TOF) of as much as 1.56 min^–1^—both in line with literature-reported values obtained for this POM under homogeneous conditions (see the SI).^17^Figure 6b provides a detailed look at the deactivation: the instant TOF value drops 10-fold within the first 10 min of illumination. This exemplary WOC experiment is representative of the unstable nature of many homogeneous photosystems^61,62^ and, in the particular case of the {Co^III^Co^II^W_11_}/[Ru(bpy)_3_]^2+^ couple, can likely be related to the rapid degradation of the photosensitizer.^17^

We further proceeded with the evaluation of the photocatalytic performance of the {Co^III^Co^II^W_11_}-APTES-TiO_2_ composite. In this set of experiments (details in the Experimental Section), the role of the photosensitizer is taken by the support TiO_2_. An optimal comparison between the homogeneous and heterogeneous WOC systems in terms of TOF values required the presence of a similar number of POM clusters in the reaction solution (e.g., on the TiO_2_ surface) in both cases. Considering the {Co^III^Co^II^W_11_} loading of 14 wt %, 0.046 μmol of POM clusters are available for WOC in a single catalytic run (details in the SI). This number corresponds well to the 20 μM {Co^III^Co^II^W_11_} solution used in the homogeneous system, which amounts to 0.04 μmol of the POMs.

Figure 6a (bottom) presents the WOC performance of the {Co^III^Co^II^W_11_}-APTES-TiO_2_ composite against that of the molecular POM solution. Several important observations can be made. In comparison with bare TiO_2_ (Figure S8) or APTES-TiO_2_ reference (that shows almost negligible WOC activity), the {Co^III^Co^II^W_11_}-APTES-TiO_2_ composite exhibits enhanced O_2_ evolution, yielding 1 μmol of O_2_ after 60 min of illumination. This result suggests two important points: first, the heterogenized POM clusters promote the reaction of interest, that is, they act as WOC co-catalysts; second, TiO_2_ generates free charge carriers able to take part in the redox reactions of interest, that is, it acts as a photosensitizer (further experiments in the SI). In addition to these—and in contrast to the homogeneous WOC case—a linear O_2_ evolution is obtained for the {Co^III^Co^II^W_11_}-APTES-TiO_2_ composite, which corresponds to a constant reaction rate. Long-term experiments further validate this point: no saturation in WOC activity is observed for at least 10 h (Figure S9), while the amount of generated O_2_ approximates a TON of 82.5, one of the highest values among other Co-based POMs tested under similar homogeneous WOC conditions.^16^

Considering the number of {Co^III^Co^II^W_11_} co-catalyst clusters on TiO_2_, the WOC performance of the {Co^III^Co^II^W_11_}-APTES-TiO_2_ can be translated to an average TOF of around 0.39 min^–1^. Figure 6b plots instant TOF values of both photosystems and shows that despite the initial TOF of the POM cluster in the solution being at least 4-fold higher, a strong stabilization effect is in place, which renders the {Co^III^Co^II^W_11_}-APTES-TiO_2_ composite a superior light-driven WOC already after 5 min of turnover conditions. The strongly improved long-term WOC performance of the heterogenized POM photosystem can be related to (i) the replacement of the molecular [Ru(bpy)_3_]^2+^ with a more stable and robust inorganic absorber, which allows for a constant photosensitization rate as well as (ii) the stabilization of the individual POM clusters on the support, which helps prevent cluster aggregation and the deactivation of their active sites.

The initially higher TOF values of the homogeneous system (1.56 min^–1^ vs 0.39 min^–1^) can be explained by the complex interplay between photoactivation and catalytic function. In the homogeneous system, we use a comparably high concentration of [Ru(bpy)_3_]^2+^ (photosensitizer-to-POM ratio of 40:1), which—in concert with a higher POM mobility in solution—accounts for more efficient light absorption and charge transfer upon collision with the POM molecules. This photosystem, however, also deactivates rapidly. In contrast, the POM clusters in the heterogeneous system are fixed in position at the APTES/TiO_2_ surface and rely on the charge carriers that are provided by the TiO_2_ photosensitizer. It is likely that either the photoexcitation in TiO_2_ or the charge transfer to the POMs are limiting factors that define the suboptimal TOF values obtained for the {Co^III^Co^II^W_11_}-APTES-TiO_2_ composite. Future studies using supports with more efficient charge excitation and transfer dynamics will be required to achieve higher WOC efficiency values.

Post-catalytic characterization of the heterogenized composite with a set of complementary techniques confirms the integrity of the {Co^III^Co^II^W_11_}-APTES-TiO_2_ attachment; however, a certain degree of leaching of the POM clusters could also be observed (Figure S10). In light of the stable WOC performance recorded for the heterogenized photosystem, we suggest that either the leaching takes place during the initial stage of the photocatalytic run, affecting only some of the clusters (possibly defined by the low denticity of APTES attachment or high coverage of the POM anions in some areas) or the detached {Co^III^Co^II^W_11_} clusters continue to contribute to the WOC activity (details in the SI).

### WOC Mechanism

**Figure 7:**
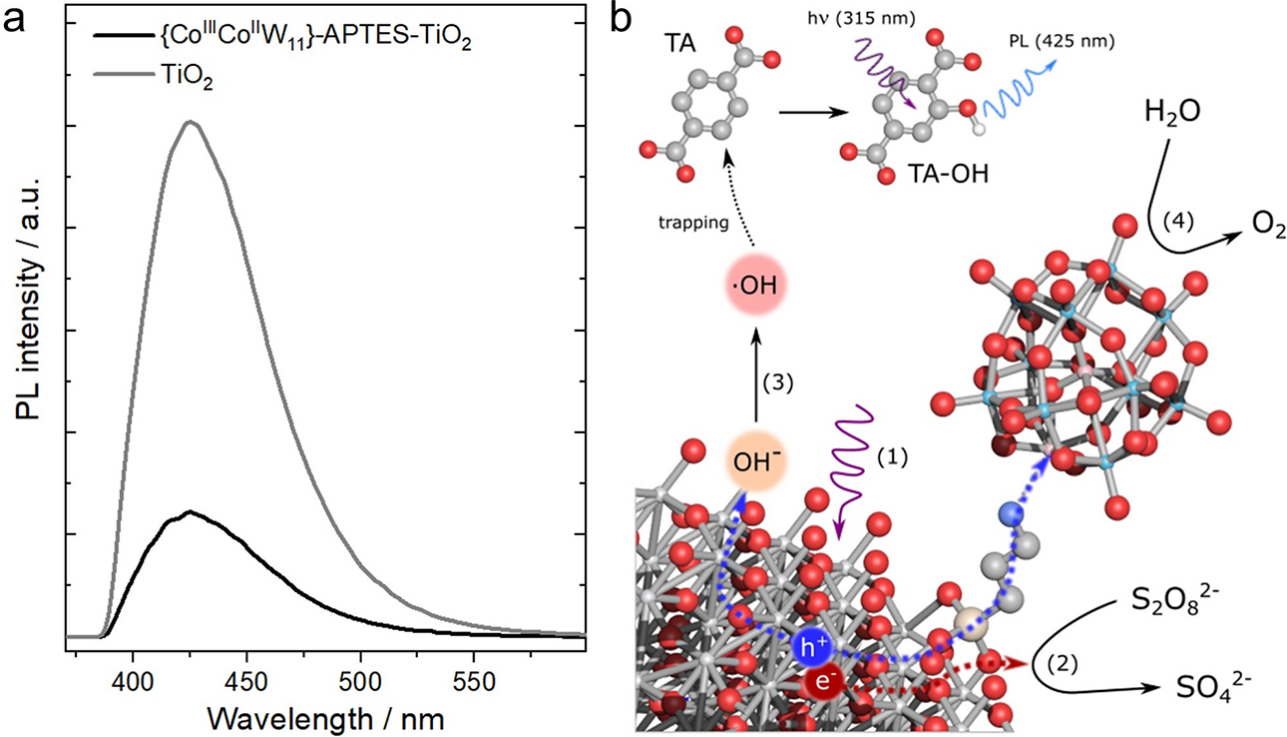
WOC mechanism and its investigation using PL spectroscopy: (a) PL spectra of supernatants of anatase and {Co^III^Co^II^W_11_}-APTES-TiO_2_ probing the PL emission of TA-OH produced via ·OH attack of terephthalic acid over the course of illumination. (b) Schematic illustrating various charge transfer processes occurring at the surface of the {Co^III^Co^II^W_11_}-APTES-TiO_2_ composite upon UV light illumination, which also demonstrates how PL can be used to probe the amount of ·OH formed during WOC reaction. The equation numbers indicate respective reactions taking place (as mentioned in the WOC Mechanism section).

The general mechanism of water oxidation on TiO_2_ surface—when in the presence of Na_2_S_2_O_8_ oxidant—can be described as shown in Figure 7b by the following reactions:
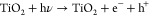
1
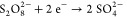
2
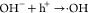
3
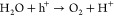
4

Water oxidation to produce O_2_ (eq 4) can take place via two major pathways:^63^ water nucleophilic attack (WNA) or the interaction of two metal-oxo entities (I2M). In the case of WNA, the Ti-oxo species undergo a nucleophilic attack of a H_2_O molecule and a subsequent proton–electron transfer (PET) to generate the O–O (peroxo) bond, whereas the I2M mechanism involves the coupling of two separate metal-oxo moieties for O–O bond formation.

To understand whether the oxidation by photogenerated holes leads to the formation of OH radicals (·OH) or O_2_ (competing steps, eqs 3 and 4), we employed photoluminescence (PL) emission spectroscopy and used terephthalic acid (TA) as the fluorescence probe that can effectively trap ·OH.^64^Figure 7b shows that the reaction of ·OH with TA forms highly fluorescent 2-hydroxyterephthalic acid (TA-OH); its quantification by means of PL thus provides an estimate of the amount of ·OH produced by the photocatalyst (further details in the Experimental Section and the SI). The PL emission studies were conducted for TiO_2_ and the {Co^III^Co^II^W_11_}-APTES-TiO_2_ composite. Figure 7a shows the TA-OH emissions from the reaction solution supernatants (with a peak centered at 425 nm). In the case of TiO_2_, this peak has a much higher (4-fold) intensity compared to that of {Co^III^Co^II^W_11_}-APTES-TiO_2_. This indicates that the generation of ·OH on the TiO_2_ surface (Figure 7b and eq 3) is suppressed in the presence of attached POM clusters, hence leading to more efficient utilization of holes for water oxidation via direct hole transfer (Figure 7b and eq 4). In the case of bare TiO_2_, ·OH generation occurs more effectively (Figure 7b and eq 3), which in turn inhibits O_2_ formation and leads to a lower WOC activity. Overall, this data suggests that the presence of the POM on the surface allows for more efficient hole extraction and utilization toward water oxidation, which manifests the role of {Co^III^Co^II^W_11_} as a co-catalyst.

## Conclusions

We report a novel photocatalytic system composed of an all-inorganic molecular Co-containing [Co^III^Co^II^(H_2_O)W_11_O_39_]^7–^ Keggin-type POM cluster immobilized onto the TiO_2_ surface using an APTES linker. The composite was thoroughly characterized with regard to its structure and composition using ATR-FTIR, powder XRD, ^29^Si-ss-NMR, XPS, ICP-MS, and TXRF to reveal POM loadings, surface coverage, and distribution as well as to probe the POM/APTES and APTES/TiO_2_ binding modes. High-resolution HAADF-STEM images were used to visualize the [Co^III^Co^II^(H_2_O)W_11_O_39_]^7–^ attachment as well as to confirm its structural integrity on the support surface. Photocatalytic WOC studies were conducted under heterogeneous and homogeneous conditions. We demonstrated that in comparison with bare TiO_2_, the composite performs as a more efficient photocatalyst for light-driven water oxidation, which manifests the role of heterogenized {Co^III^Co^II^W_11_} as a WOC co-catalyst. In contrast to the homogeneous WOC performance of this POM, the heterogenized clusters showed strongly improved long-term activity without any apparent deactivation for at least 10 h. This stable WOC performance is achieved without the presence of any external photosensitizer, which implies that TiO_2_ not only acts as a support for POM anchoring but also as a stable light absorber. Furthermore, we uncovered the WOC mechanism utilizing PL spectroscopy. Our data revealed that the more active WOC performance of the {Co^III^Co^II^W_11_}-APTES-TiO_2_ over TiO_2_ is related to the effective hole extraction by the POM cluster, which promotes charge separation and allows for a more efficient H_2_O oxidation by direct hole attack at the POM site. With the combination of the stable photocatalytic WOC performance with the ability for catalyst recovery, this novel photocatalytic system represents an example of an effective light-driven WOC that combines the advantages of its heterogeneous and homogeneous counterparts. We envision future studies aiming to optimize the process of photosensitization and implement visible-light active supports, which will maximize the benefits of this photosystem.

## Supporting Information Available

TON-TOF calculations; description of characterization techniques; characterization details for {Co^III^Co^II^W_11_}; APTES-TiO_2_ and {Co^III^Co^II^W_11_}-APTES-TiO_2_ (including FTIR, XRD, XPS, TGA, and additional STEM/EDS data); calculation of the surface coverage; discussion of the WOC activity trends; additional WOC experiments; post-catalytic characterization (XPS, TXRF, and FTIR); mechanistic studies (PDF)
