# Supplementary material for: Immobilization of a [CoIIICoII(H2O)W11O39]7– Polyoxoanion for the Photocatalytic Oxygen Evolution Reaction
Source: ACS Mater Au. 2022 May 25;2(4):505–15. doi: 10.1021/acsmaterialsau.2c00025 (PMC9284608; doi:10.1021/acsmaterialsau.2c00025)
Supplement: Supplementary file 1 — mg2c00025_si_001.pdf [file mg2c00025_si_001.pdf]

Supporting information

Immobilization of a $[Co^{III}Co^{II}(H_2O)W_{11}O_{39}]^{7-}$ polyoxoanion for photocatalytic oxygen evolution reaction

*Sreejith P. Nandan[1], Nadiia I. Gumerova[2]**, Jasmin S. Schubert[1], Hikaru Saito[3], Annette Rompel[2], Alexey Cherevan[1]** and Dominik Eder[1]*

[1] *TU Wien, Institute of Materials Chemistry, Getreidemarkt 9/BC/02, 1060 Vienna, Austria; www.imc.tuwien.ac.at*
[2] *Universität Wien, Fakultät für Chemie, Institut für Biophysikalische Chemie, Althanstraße 14, 1090 Vienna, Austria; www.bpc.univie.ac.at*
[3] *Institute for Materials Chemistry and Engineering, Kyushu University, 6-1 Kasugakoen, Kasuga, Fukuoka 816-8580, Japan*

** correspondence to:*
nadiia.gumerova@univie.ac.at
alexey.cherevan@tuwien.ac.at

Contents

1. Experimental section 3
2. Characterization techniques 3
3. Characterization of {$Co^{III}Co^{II}W_{11}$} 6
4. Characterization of APTES-TiO_2 8
5. Grafting of {$Co^{III}Co^{II}W_{11}$} onto APTES-TiO_2 9
6. Characterization of {$Co^{III}Co^{II}W_{11}$}-APTES-TiO_2 10
7. Monolayer adsorption model for {$Co^{III}Co^{II}W_{11}$}@TiO_2 15
8. Comparison of OER activity values with those reported previously 16
9. Additional OER experiments 16
10. Post-catalytic characterization 17
11. PL investigation of the WOC mechanism 19
12. References 20

1. Experimental section

TON-TOF calculations

The turnover number (TON) is calculated as:

$$TON = \frac{number\ of\ moles\ of\ O_2\ produced}{number\ of\ moles\ of\ catalytic\ active\ sites}$$

The number of moles of catalytic active sites is assumed to be equal to the number of POMs present in the WOC reaction solution.

The turnover frequency (TOF) is defined as:

$$TOF\ (min^{-1}) = \frac{TON}{time\ (min)}$$

The instant TOF is elucidated by taking the first derivative (*d*) of the plot between TON and time (min)

$$Instant\ TOF\ (min^{-1}) = \frac{d\ (TON)}{d\ (time)}$$

In the homogeneous WOC: Considering 20 µM POM is taken for the WOC, and the total volume of the reaction solution is 2 ml, the number of solubilized POMs is calculated to be 0.040 µmol.

In the heterogeneous WOC: Based on the amount of photocatalyst (1 mg) used in every run and POM loadings (14 wt%), the number of moles of immobilized POMs can be estimated to be 0.046 µmol.

2. Characterization techniques

Attenuated Total Reflectance-Infrared (ATR-IR) spectroscopy: All Fourier-transform IR (FT-IR) spectra were recorded on a Bruker Tensor 27 (Ettlingen, Germany) IR Spectrometer equipped with an ATR unit with a single-reflection diamond.

Powder X-ray diffraction (XRD): The diffraction patterns were recorded using an XPERT II: PANalytical XPert Pro MPD (Θ–Θ diffractometer). The sample was irradiated using a Cu X-ray source (8.04 keV, 1.5406 Å) after placing it on a Si sample holder. Bragg–Brentano Θ/Θ-diffractometer geometry was employed, and the signals were acquired from 5 to 80 degrees with a semiconductor X'Celerator (2.1°) detector.

Thermogravimetric Analysis (TGA): TGA measurements were done on a PerkinElmer Thermogravimetric Analyser, TGA 8000. The samples were kept in an Al_2O_3 crucible and were heated up with a ramp rate of 5 °C/min under air and N_2, ranging from 30 °C to 700 °C. These results were then used to determine the number of crystal waters present in $\{Co^{III}Co^{II}W_{11}\}$.

Electrospray-ionization mass spectrometry (ESI-MS): The analysis was performed with an ESI−Qq−oaRTOF supplied by Bruker Daltonics Ltd. The measurements were carried out in H_2O and in a mixture of $CH_3CN/MeOH/H_2O$, collected in negative ion mode and with the spectrometer calibrated with the standard tune−mix to give an accuracy of better than 5 ppm in the region of m/z 100−1900. The Bruker Daltonics Data Analysis software was used to analyze the results.

X-ray Photoelectron Spectroscopy (XPS): A custom-built SPECS XPS-spectrometer equipped with a monochromatized Al-K_α X-ray source (µ 350) and a hemispherical WAL-150 analyzer with an acceptance angle of 60° was used to investigate the chemical composition of the samples using XPS. Pass energies of 100 eV and 30 eV and energy resolutions of 1 eV and 100 meV were used for survey and detail spectra, respectively (excitation energy: 1486.6 eV, beam energy and spot size: 70 W onto 400 µm, angle: 51° to sample surface normal, base pressure: 5×10^{-10} mbar, pressure during measurements: 2×10^{-9} mbar). The data analysis was performed using CASA XPS software, employing transmission corrections (as per the instrument vendor's specifications), Shirley and Sh Tougaard backgrounds and Scofield sensitivity factors. The data were calibrated with the adventitious carbon method to 284.8 eV.

^{29}Si solid-state NMR spectroscopy: ^{29}Si magic-angle spinning (MAS) NMR spectra were recorded on a Bruker FT-NMR spectrometer Avance Neo 500 MHz (Bruker, Rheinstetten, Germany) at the resonance frequency of 79.49 MHz for ^{29}Si using the cross-polarization (CP), magic-angle spinning (MAS), and a high-power ^{1}H decoupling. The powder samples were placed in a pencil-type zirconia rotor of 4.0 mm outer diameter. The spectra were obtained at a spinning speed of 8 kHz (4 µs 90° pulses), a 8-ms CP pulse, and a recycle delay of 4 seconds. The Si signal of tetramethylsilane (TMS) at 0 ppm was used as the reference of ^{29}Si chemical shift.

Total reflection X-ray fluorescence (TXRF) spectroscopy: TXRF was performed to analyze the elemental composition of the reaction solutions and solids with detection limits in the ppb range. Stoichiometric ratios between various elements in $\{Co^{III}Co^{II}W_{11}\}$, and their loading amounts on TiO_2 can be determined. As Si-based (quartz) sample holders were used, quantifying APTES amounts on TiO_2 was not feasible. All the TXRF measurements were carried out using Atomika 8030C X-ray fluorescence analyzer (Atomika Instruments GmbH, Munich, Germany). This spectrometer operates with a total reflection geometry using an energy-dispersive Si(Li)-detector, and the measurements were done with monochromatized Mo-Kα excitation mode at 50 kV and 47 mA, for 100 s live time. The solid samples (1 mg) were fixated on quartz reflectors with the help of 5 µL, 1% polyvinyl alcohol (PVA) solution, which was then dried for 5 min on a hot plate. The amount of Ti was set to 100% and relative amounts of the elements (wt%) of interest were acquired. For liquid samples, a fixed concentration (10 ppm) of Cr was added as an internal standard for the quantification of elements. The solution was vortexed, dropped (5 µL) onto quartz reflectors, dried on a hot plate, and the obtained film was then analyzed.

Transmission electron microscopy (TEM): The images were obtained using a FEI TECNAI F20 transmission electron microscope equipped with a field emission gun in bright field mode using 200 kV acceleration voltage. The sample was prepared from powdered samples directly on a copper holey carbon-coated grid (Plano, 200 mesh). *Energy-dispersive X-ray spectroscopy (EDS)* was performed using the scanning TEM (STEM) mode to obtain elemental maps.

High-angle annular dark-field (HAADF) STEM: The images and EDS maps were obtained using Titan Cubed G2 60-300 (TEM/STEM, FEI Co., now Thermo Fisher Scientific) operated at 300 kV. This microscope has an aberration corrector for STEM (DCOR, CEOS), four-quadrant windowless super-X SDD (silicon drift detector) system. The probe current was ~60 pA for STEM observation as well as EDS. The convergence semi-angle of the electron probe was 18 mrad. The typical probe diameter was less than 0.1 nm. Forward scattered electrons of an angular range from 38 to

184 mrad were detected by a HAADF detector for STEM imaging. For structural characterization by HAADF-STEM and EDS, the sample was dispersed on a grid with an amorphous carbon supporting film. The sample surface was additionally covered with an amorphous carbon film by evaporation for suppressing damage by electron beam irradiation.

N_2 physisorption and Brunauer-Emmett-Teller (BET) analysis: Physisorption measurements were carried out on a Micromeritics ASAP 2020 instrument (Micromeritics GmbH). The degassing protocol was set to 150 °C overnight and the isotherms were obtained using nitrogen at 77 K as adsorbate. The specific surface areas were then calculated using BET analysis.[1]

Inductively coupled plasma mass spectrometry (ICP-MS): Elemental analyses were performed in aqueous solutions containing 2% HNO_3 using inductively coupled plasma mass spectrometry (PerkinElmer Elan 6000 ICP MS) for Co, W and Ti and atomic absorption spectroscopy (PerkinElmer 1100 Flame AAS) for K. Standards were prepared from single-element standard solutions of concentration 1000 mg/L (Merck, Ultra Scientific and Analytika Prague).

Photoluminescence (PL) spectroscopy: PL steady-state measurements were performed using PicoQuant FluoTime 300 spectrophotometer. Xe arc lamp (300 W power) was the excitation source, coupled with a double-grating monochromator. The detection system is comprised of a PMA Hybrid 07 detector along with a high-resolution double monochromator. The excitation wavelength utilized for all the steady-state measurements was 315 nm (3.94 eV photon energy). The data was collected and later fitted using EasyTau2 software.

3. Characterization of {Co^{III}Co^{II}W$_{11}$}

Figure S1a shows the ATR-FTIR spectrum of {Co^{III}Co^{II}W$_{11}$} in the POM fingerprint region, validating the presence of W=O and W-O-W bands at 947 and 880 cm^{-1} respectively,[2] along with peaks at 656, 737 and 771 cm^{-1}, indicative for Keggin structure. The detailed Co 2p XPS spectrum shows 2 peaks with binding energies (BE) of 781.6 and 796.9 eV, along with 2 shoulder peaks at higher binding energies of 787.0 and 803.9 eV respectively. Given that bond-valence sum (BVS) calculations from single-crystal XRD confirm a central Co(III),[2] and the overall charge of POM calculated from ICP-MS is -7, the charge on the peripheral Co is +2. Thus, XPS confirms the presence of both Co(II) and Co(III) in {Co^{III}Co^{II}W$_{11}$}. Powder XRD matches well with literature[2] and simulated pattern from single-crystal XRD, thus confirming the identity and structural purity of {Co^{III}Co^{II}W$_{11}$}.

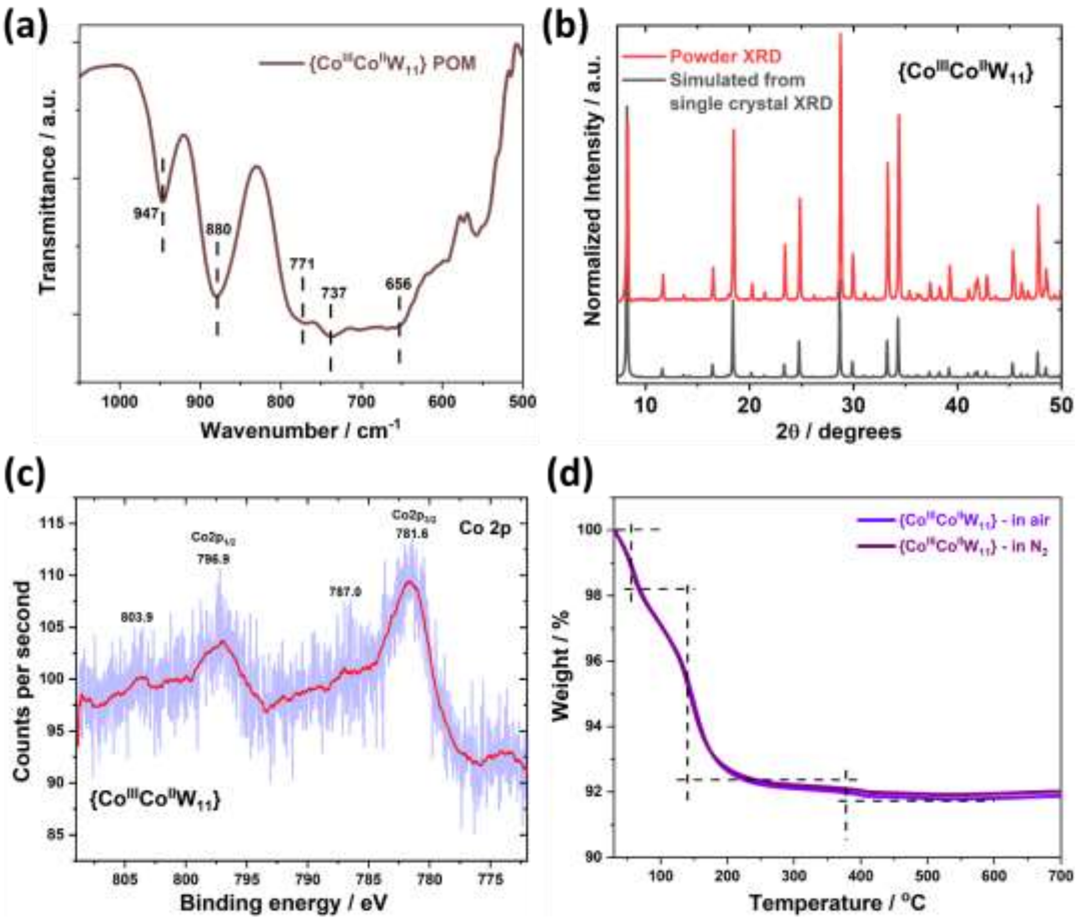

Figure S1: Characterization of {Co^{III}Co^{II}W$_{11}$} POM: (a) ATR-FTIR showing the presence of W=O and W-O-W at 947 and 880 cm^{-1} respectively, (b) powder XRD of {Co^{III}Co^{II}W$_{11}$} compared to its simulated pattern from single-crystal XRD, (c) XPS – Co 2p showing the peaks at 781.6 and 796.9 eV, and their higher-energy satellite peaks at 787.0 and 803.9 eV, and (d) TGA under air and N_2 atmospheres from 30 till 700 °C.

As shown in Table S1, TGA shows that a mass loss of 7.9 wt% occurs until a temperature of 265 °C, and correspondingly, the number of crystal H_2O was elucidated to be 14. In the temperature regime from 265 until 500 °C, an additional loss of one H_2O molecule is observed, which would correspond to the H_2O coordinated to Co^{II} within the polyanion.

Table S1: TGA of $\{Co^{III}Co^{II}W_{11}\}$ – in air and N_2.

Step	***Temperature range (°C)***	***Mass loss (%)***	***Number of crystal water***
1	*30 – 75*	*2.0*	***3.5***
2	*76 – 265*	*5.9*	***10.5***
3	*266 – 500*	*0.4*	***1***

ICP-MS confirmed the elemental composition of $\{Co^{III}Co^{II}W_{11}\}$ with a stoichiometric ratio between Co: W: K obtained as 2.1: 11: 7.4 for a theoretical ratio of 2: 11: 7. It is also confirmed from the number of counter cations that the overall charge on the polyanion is -7. Additionally, TXRF is employed to corroborate the elemental composition of $\{Co^{III}Co^{II}W_{11}\}$, as shown in Table S2.

Table S2: Elemental composition of $\{Co^{III}Co^{II}W_{11}\}$ – using both ICP-MS and TXRF.

Stoichiometric ratios – $K_7[Co^{III}Co^{II}(H_2O)W_{11}O_{39}]$			
Technique used	***Co***	***K***	***W***
ICP MS – weight ratios	*5.1*	*11.9*	*83.0*
TXRF – weight ratios	*4.7*	*11.3*	*84.0*
ICP MS – atomic ratios	***2.1***	***7.4***	***11.0***
TXRF – atomic ratios	***1.9***	***7.0***	***11.0***
Theoretical atomic ratios	***2.0***	***7.0***	***11.0***

4. Characterization of APTES-TiO_2

Figure S2a shows the XPS survey spectrum of APTES-TiO_2 confirming the presence of Si 2p, N 1s and Ti 2p and O 1s. The Ti 2p spectrum (Figure S2b) shows no change in peak shape and position in comparison with bare TiO_2, which confirms Ti(IV) in APTES-TiO_2. The N 1s XPS spectrum (Figure 2d) indicates the presence of free NH_2 and H-bonded or protonated amino groups. The presence of a N-O-Ti or N-Ti bond can be excluded, as this would appear at much higher and lower binding energies (BEs), respectively.[3,4]

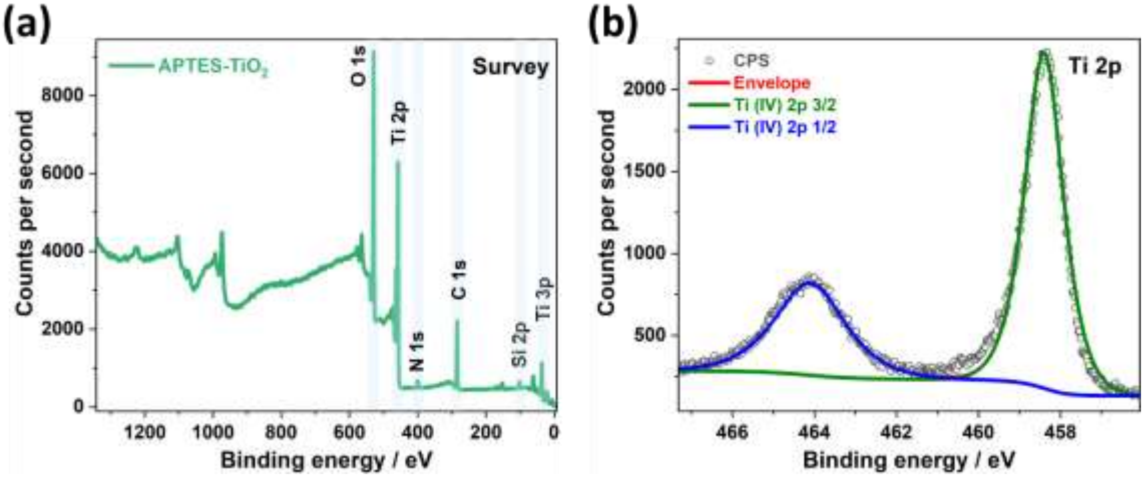

Figure S2: XPS of APTES-TiO_2: (a) Survey spectrum indicating the presence of elements of both APTES and TiO_2, and (b) Ti 2p spectrum demonstrating the presence of Ti(IV) in APTES-TiO_2.

Table S3: Loading (wt%) of APTES onto TiO_2 calculated from ICP-MS measurements.

Technique used	*Si***	*Ti***
ICP-MS – elemental weight ratios	*1.3*	*52.6*
ICP-MS – loading (wt%)	***11.7***	***100***

* Loading of x wt% implies x mg of *APTES* is attached to 100 mg of TiO_2.
** Calculation done based on Si/Ti and Ti/Ti experimental signal values.

Table S3 shows ICP-MS data which give the loading amounts of APTES onto TiO_2 surface. From the Si amount, the loading amount of APTES on TiO_2 is calculated to be 11.7 wt%.

5. Grafting of $\{Co^{III}Co^{II}W_{11}\}$ onto APTES-TiO_2

Choice of the POM for the attachment

The binding interaction of terminal oxygen ions O_t with the d^0 addenda metal (e.g. fully oxidized W^{6+}, Mo^{6+} or V^{5+}) in POMs leads to a six-electron donation (one σ and two π d-p bonds) to the metal center forming a very strong bond,[5] and, therefore, O_t ions in intact POM structures are relatively inert. When O_t atoms are replaced by an amine (NR_3), imine (R-N=CR_2) or amide (R-$CONH_2$), the resulting amino functionalization is considered largely as a σ-bond with weak π-contribution, and therefore less stable than a pronounced multiple bond interaction. Thus, only POM structures with suitable addenda centers that do not require further stabilization by electron donation through the ligand were found with this kind of substitution. Such addenda atoms require an increased electron density as provided by either reduction (e.g. V^{IV} in $[Fe^{II}V^{IV}{}_6O_6\{(OCH_2CH_2)_3N\}_6]^{2+}$)[6] or in lacunary anions addenda atoms with charged cis-dioxo sites (e.g. $[P^{V}Mo^{VI}{}_9O_{34}]^{9-}$ in A-α-$[(P^{V}Mo^{VI}{}_9O_{31})(NC_5H_5)_3]^{3-}$).[7] While the parent $[PW_{12}O_{40}]^{3-}$ compound is unable to undergo the proposed attachment with APTES moieties, in the case of transition-metal substituted Keggin type POMs such as in the selected $\{Co^{III}Co^{II}W_{11}\}$ cluster, a water molecule terminally coordinated to the transition metal ion can indeed be replaced by many other ligands, including amines, given the high Co affinity to -NH_2 with stronger Co−N than Co−O bonding.[8] A close look into the literature confirms that the coordination of transition metal ions to alternative ligands has been demonstrated multiple times – in pure water and aqueous solutions – both during targeted synthesis[9–13] and in real systems, for example, in the formation of bonds with amino acid residues during coordination to proteins.[14,15]

Confirmation of the $\{Co^{III}Co^{II}W_{11}\}$ / amine interactions

The possibility of aqua ligand displacement for transition metal substituted POMs in aqueous media by ligands such as pyridines or ammonia was proposed and proven by Baker and Figgis way back in 1970.[16] Following the procedure described by Johnson and Stein,[17] we mixed $\{Co^{III}Co^{II}W_{11}\}$ with n-butylamine (Figure S3) to observe the expected binding. In the POM spectrum, the band at 535 nm can be assigned to a $^4T_{1g}(P) \leftarrow {}^4T_{1g}(F)$ transition typical for an octahedral Co^{II} with six oxygen ligands. The addition of n-butylamine results in the displacement of coordinated water by the organic ligand and leads to a lowering of the symmetry of the Co^{II} site and subsequent splitting of the $^4T_{1g}(P)$ band into E_g and A_{2g} components, resulting in a new absorbance observed between 550 and 730 nm (max at 640 nm). The data thus confirms the possibility of the POM grafting onto the amine moiety, which is in line with the proposed attachment model.

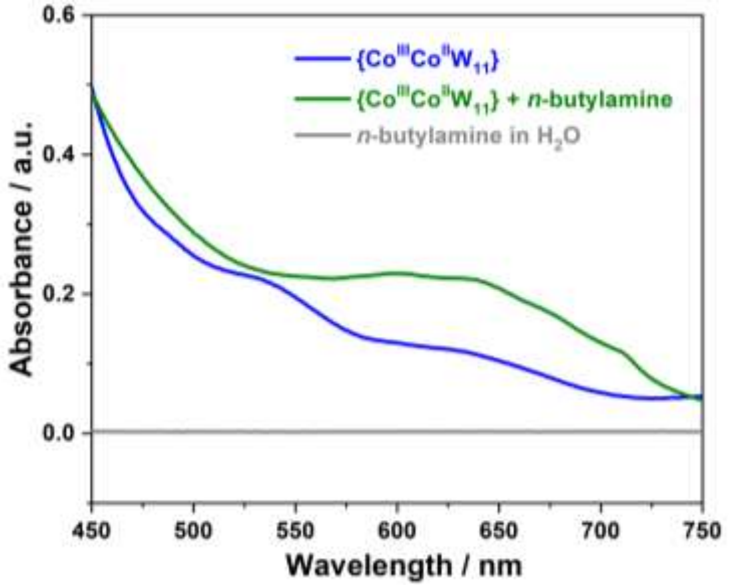

Figure S3: Solution UV−vis spectra for 1 mM {$Co^{III}Co^{II}W_{11}$} (blue), n-butylamine aqueous solution (25 μL in 975 μL of water, grey) and 1mM {$Co^{III}Co^{II}W_{11}$} + n-butylamine (25 μL, green). The new absorption peaks with a maximum at around 640 nm is due to the coordination of the amine to the Co^{II} center.

6. Characterization of {$Co^{III}Co^{II}W_{11}$}-APTES-TiO_2

STEM/EDX

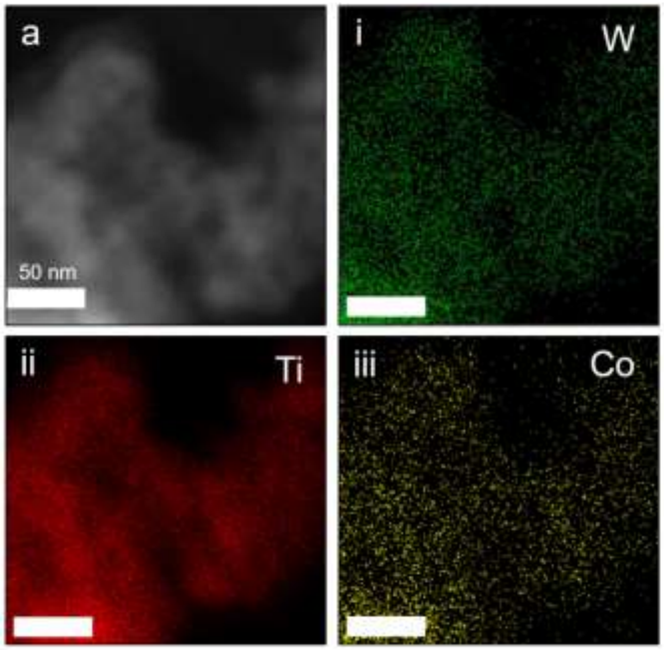

Figure S4: STEM-EDS data of {$Co^{III}Co^{II}W_{11}$}-APTES-TiO_2 composite showing (a) TEM image and elemental maps of (i) W, (ii) Ti, and (iii) Co. Scale bar corresponds to 50 nm. The maps confirm the homogeneous incorporation of the POM clusters over the sample volume.

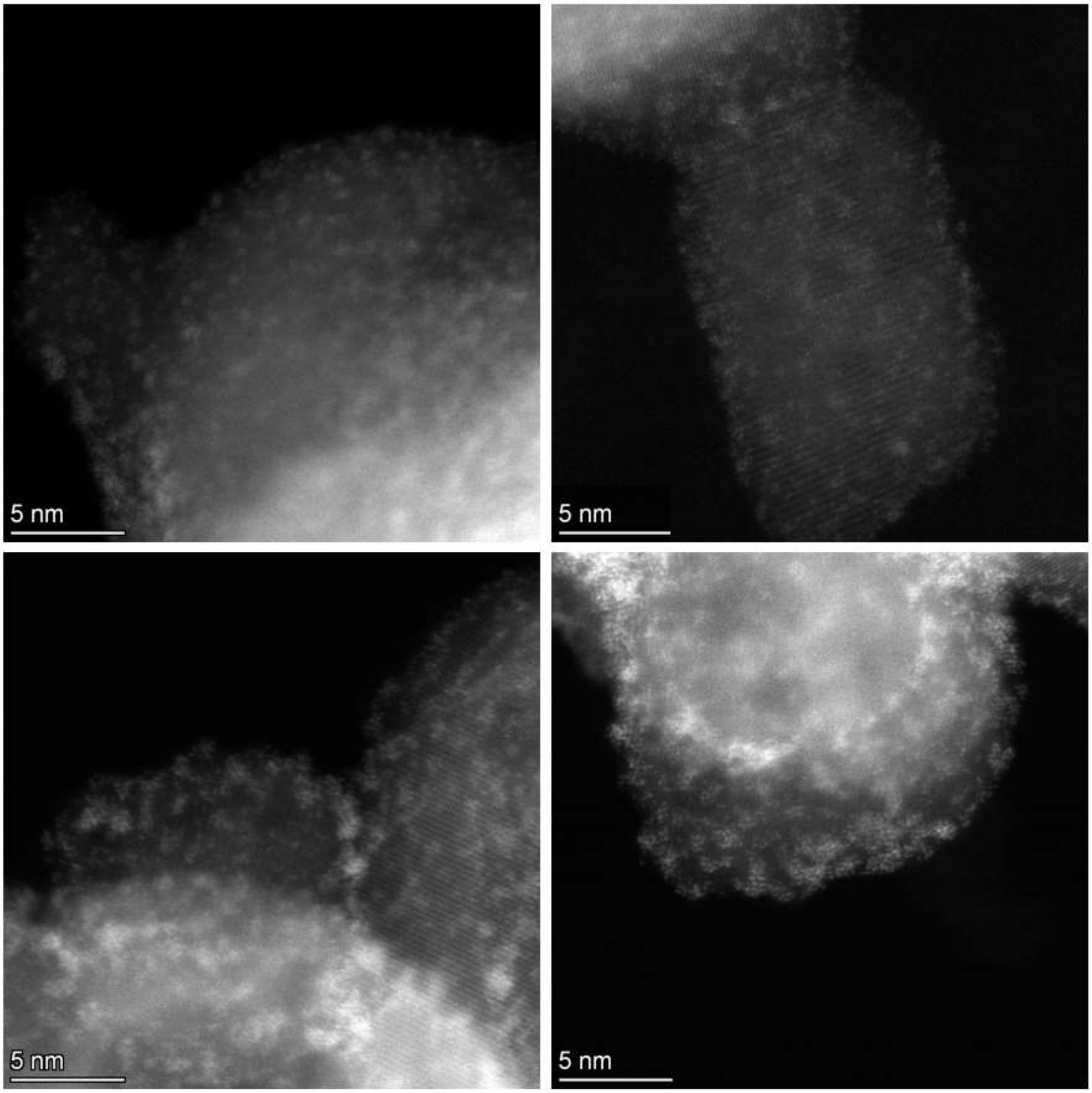

Figure S5: Additional high-resolution HAADF-STEM images of $\{Co^{III}Co^{II}W_{11}\}$-APTES-$TiO_2$ composite showing decoration of TiO_2 nanoparticles with evenly distributed $\{Co^{III}Co^{II}W_{11}\}$ clusters.

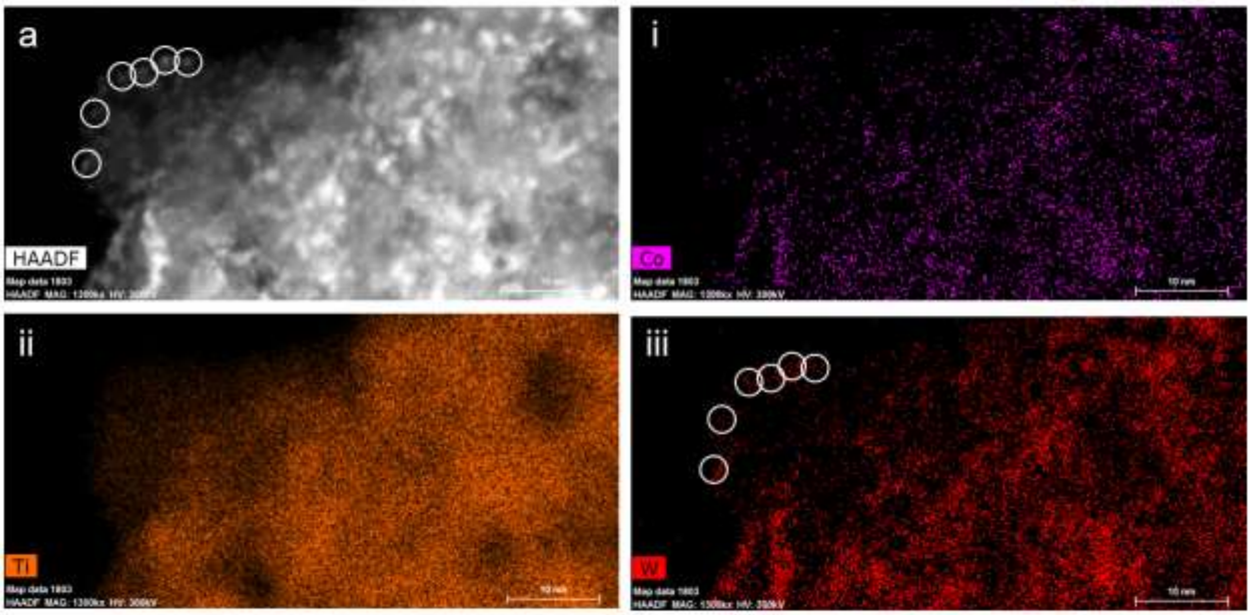

Figure S6: STEM-EDS elemental maps featuring the distribution of (i) Co, (ii) Ti, and (iii) W atoms. Areas of high W concentration (in iii) correspond well to those featuring a collection of bright spots observed in STEM (in a).

XPS

Figure S7a shows the survey spectrum of $\{Co^{III}Co^{II}W_{11}\}$-APTES-$TiO_2$ confirming the presence of Co 2p, W 4f, Si 2p, N 1s, Ti 2p and O 1s (elements expected from the composition of the sample). It also indicates that W 4f and Ti 3p signals could overlap in the 32 – 40 eV region, which can complicate the analysis of W 4f edge. The detailed W 4f XPS spectra (Figure S7b) of $\{Co^{III}Co^{II}W_{11}\}$-APTES-$TiO_2$, $\{Co^{III}Co^{II}W_{11}\}$ and TiO_2 clearly shows that there is a small shift in peak position (approx. 0.5 eV) towards lower BEs in the case of the composite, which is attributed to the occurrence of Ti 3p. The Ti 2p spectrum (Figure S7c) confirms the presence of Ti(IV), and the Si 2p spectrum (Figure S7d) confirms the presence of Si(IV) in $\{Co^{III}Co^{II}W_{11}\}$-APTES-$TiO_2$, similar to the case of APTES-TiO_2. The Co 2p (Figure S7e) spectrum has a poor signal-to-noise ratio in the composite, and even in the case of the as-prepared $K_7[Co^{III}Co^{II}(H_2O)W_{11}O_{39}]\cdot 14H_2O$ salt. This can be due to the fact that only two Co atoms are present in the $\{Co^{III}Co^{II}W_{11}\}$ cluster, and also given that Co is a lighter element compared to the surrounding W atoms. So, the interpretation of the Co signal in terms of the expected Co-N interaction and the modification of the chemical environment of Co can not be made reliably.

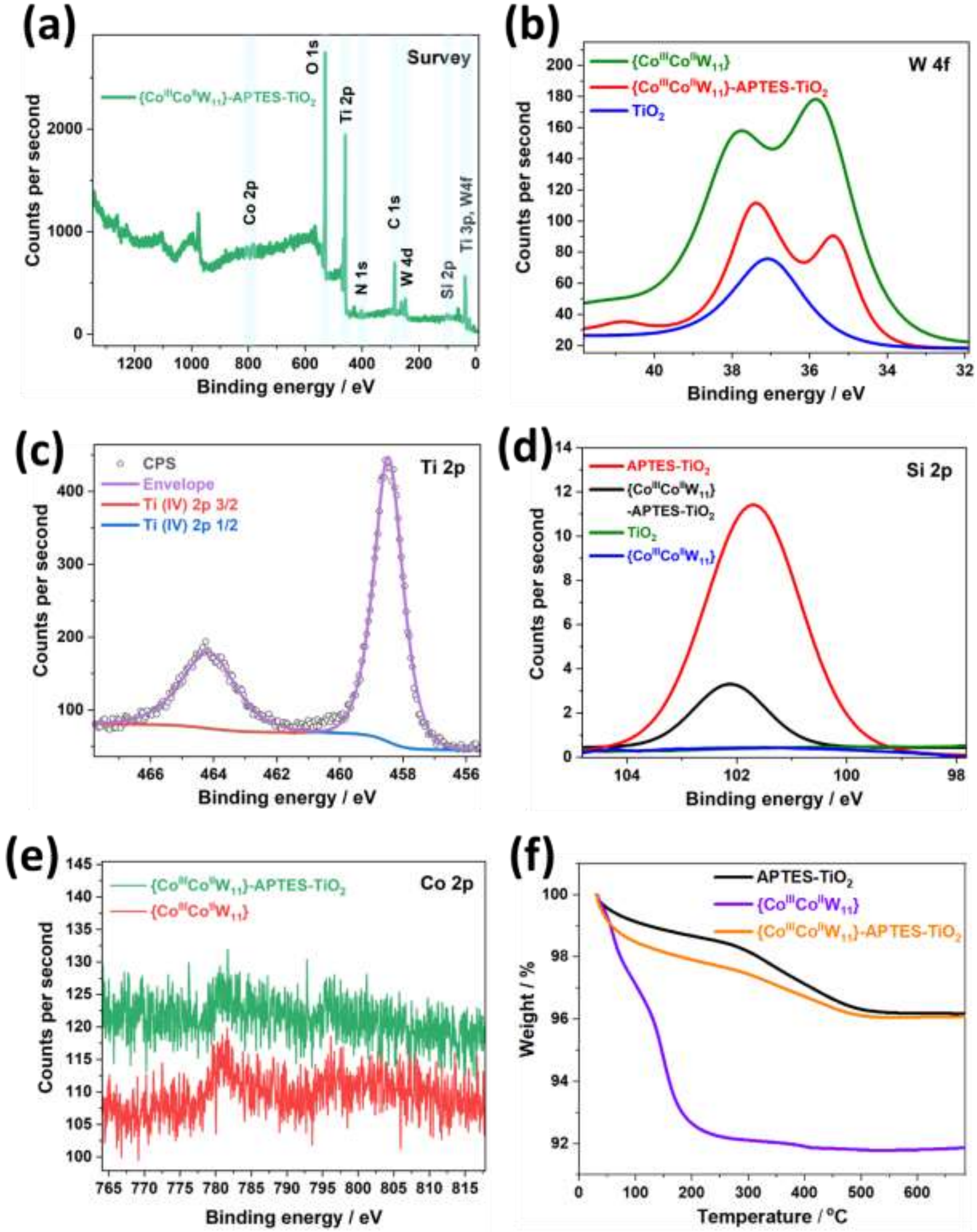

Figure S7: XPS and TGA of {$Co^{III}Co^{II}W_{11}$}-APTES-TiO_2: (a) XPS survey spectrum indicating the presence of all the elements from [$Co^{III}Co^{II}W_{11}$], APTES and TiO_2; (b) XPS – W 4f compared with individual constituents, {$Co^{III}Co^{II}W_{11}$} and TiO_2; (c) XPS – Ti 2p showing presence of Ti(IV); (d) XPS – Si 2p compared with APTES-TiO_2, TiO_2 and [$Co^{III}Co^{II}W_{11}$]; (e) XPS – Co 2p compared with [$Co^{III}Co^{II}W_{11}$]; and (f) TGA of the composite compared with APTES-TiO_2, and {$Co^{III}Co^{II}W_{11}$} under ambient air atmosphere.

Elemental analyses

Both ICP-MS and TXRF were utilized to confirm the loading amounts of {$Co^{III}Co^{II}W_{11}$} on TiO_2, as shown in Table S4. Si cannot be quantified reliably using TXRF spectroscopy, as Si-based sample holders are utilized. Hence, the Si values are only estimated from ICP MS, as shown below.

Table S4: Loading (wt%) of {$Co^{III}Co^{II}W_{11}$} onto APTES-TiO_2 calculated from ICP-MS and TXRF measurements.

Technique used	***Co*****	***W*****	***Si*****
ICP-MS – elemental weight ratios	*0.9*	*15.5*	*1.5*
TXRF – elemental weight ratios	*0.7*	*16.0*	*-*
ICP-MS – POM loading (wt%)*	***12.9***	***14.1***	***7.3***
TXRF – POM loading (wt%)*	***11.2***	***14.5***	***-***

* Loading of x wt% implies x mg of *{$Co^{III}Co^{II}W_{11}$}* is attached to 100 mg of APTES-TiO_2.
** Calculation done based on Co/Ti and W/Ti and Si/Ti experimental signal values. The loading values based on W/Ti ratios more reliable considering the much higher mass and the atomic ratio of W to that of Co in {$Co^{III}Co^{II}W_{11}$}.

The sample was also investigated using scanning transmission electron microscopy - electron dispersive X-ray spectroscopy (STEM-EDS), several areas with a scale of 200x200 nm were analyzed, and elemental maps based on W signals from three representative areas were used to calculate the {$Co^{III}Co^{II}W_{11}$} loadings. Table S5 shows an average {$Co^{III}Co^{II}W_{11}$} loading of 16.1 wt% on TiO_2, which fits well with the weight percentages obtained from ICP-MS and TXRF calculations.

Table S5: Loading (wt%) of {$Co^{III}Co^{II}W_{11}$} onto APTES-TiO_2 calculated from STEM-EDS measurements.

Technique used	***W**, L line*******
EDS - area 1	*12.3*
EDS - area 2	*8.2*
EDS - area 3	*7.3*
Average	*9.3*
EDS - loading (wt%)	***16.1***

* Loading of x wt% implies x mg of *{$Co^{III}Co^{II}W_{11}$}* is attached onto 100 mg of APTES-TiO_2.
** Calculation done based on W/Ti experimental wt% values.

7. Monolayer adsorption model for $\{Co^{III}Co^{II}W_{11}\}$@$TiO_2$

Brunauer-Emmett-Teller (BET) analysis[1] on N_2-physisorption data was utilized and the surface area of TiO_2 was elucidated to be 78.63 m^2/g. Considering a monolayer coverage model of POMs on TiO_2, the maximum possible loading of $\{Co^{III}Co^{II}W_{11}\}$ onto TiO_2 was determined to be 20.8 wt%. Here, the amount of APTES linkers is ignored, and the molar mass of $\{Co^{III}Co^{II}W_{11}\}$ was calculated without taking into consideration the waters of crystallization, as they are only relevant for solid-state POM crystals and not for molecular clusters of POMs on TiO_2. The water molecules that could be physisorbed are also not considered as their interactions with POM and APTES are intrinsically dynamic in nature. For calculating the amount of APTES, only ICP-MS was employed as TXRF spectroscopy used Si-based sample holders, which makes the quantification of APTES unreliable.

Now, considering the APTES linkers, with a footprint[18] of 0.27 nm^2, a maximum loading for a monolayer of APTES on anatase has been elucidated to be 11 wt%. Given that 7.3 wt% APTES is loaded on TiO_2 in the composite, it can be safely assumed that there is some available TiO_2 surface available for $Na_2S_2O_8$ adsorption.

To help visualize the composition of the composite, let's consider one anatase nanoparticle and understand the coverage of both APTES and $\{Co^{III}Co^{II}W_{11}\}$ onto its surface. Considering the surface area of TiO_2 to be 78.6 m^2/gm, the density of anatase to be 3.9 g/cm^3, and the loadings of APTES and $\{Co^{III}Co^{II}W_{11}\}$ in the composite are measured to be 7.3 and 14 wt%, the numbers have been calculated as 3053 APTES linkers and 415 $\{Co^{III}Co^{II}W_{11}\}$ clusters per anatase particle.

8. Comparison of OER activity values with those reported previously

In contrast to Song *et al.*[2] who reported an insoluble substance being formed at $\{Co^{III}Co^{II}W_{11}\}$ concentrations higher than 15 µM, we didn't observe any formation of such insoluble substance when using 20 µM catalyst. Besides, under our stated conditions (see Experimental section) we obtained a maximum OER activity at 20 µM $\{Co^{III}Co^{II}W_{11}\}$ – concentration which was chosen to calculate and compare TON/TOF values to the data reported by Song *et al.* Even though the authors have used a similar WOC reaction system with 1 mM $Ru[bpy]_3Cl_2$ as the photosensitizer (PS) and 5 mM $Na_2S_2O_8$ as a sacrificial agent, unlike our system (reaction volume of 2 mL and pH value of 8), they have conducted WOC experiments at pH 9 with a reaction volume of 18 mL. Compared to our activities, the authors have obtained a higher TON of 32 (11.5 µmol O_2), however, it must be noted that the O_2 evolution profiles got saturated quicker under turnover conditions (after 8 min), whereas our homogenous WOC system generated O_2 continuously for about 30 min before reaching the saturation point.

9. Additional OER experiments

Reference WOC experiments have been conducted to validate our setup and elaborate on several important points: (a) APTES-TiO_2 displayed negligible WOC activity, which can be associated with charge-trapping by APTES monolayer; (b) WOC experiments with $\{Co^{III}Co^{II}W_{11}\}$-APTES-$TiO_2$ using visible light (445 nm) illumination also yielded negligible activity, which confirms the role of UV-active TiO_2 as a photosensitizer; (c) The OER activity of $\{Co^{III}Co^{II}W_{11}\}$-APTES-$TiO_2$ was compared with that of anatase; despite a much higher O_2 evolution can be seen with neat TiO_2 compared to the APTES-TiO_2, the presence of $\{Co^{III}Co^{II}W_{11}\}$ POM did result in a considerable increase in WOC rate (Figure S8).

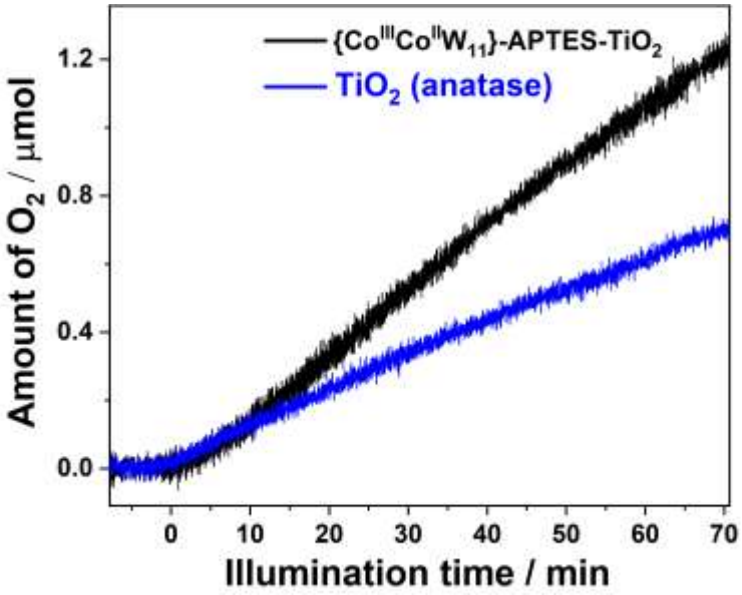

Figure S8: Comparison of WOC activity of $\{Co^{III}Co^{II}W_{11}\}$-APTES-$TiO_2$ with TiO_2.

In another series of experiments, a physical mixture of TiO_2 and {$Co^{III}Co^{II}W_{11}$} (taken in corresponding proportions to emulate 14.5 wt% POM loading) was tested yielding the amount of O_2 that was between that of neat TiO_2 and the {$Co^{III}Co^{II}W_{11}$}-APTES-TiO_2 composite. The fact that the presence of the POM clusters in the reaction solution did improve the activity of TiO_2 (although only by 15%) suggests that TiO_2 and POM species can communicate in the solution, which corresponds to the scenario *(a)* described in SI Section 10. This experiment further demonstrates the importance of the APTES-mediated POM attachment to the TiO_2 surface.

Further experiments were conducted to confirm the long-term OER stability of the {$Co^{III}Co^{II}W_{11}$}-APTES-TiO_2 composite. An exemplary plot is shown (Figure S9). Overall, we see no saturation of WOC activity even after 10 hours of illumination. The instant TOF values (can be seen as a slope of the O_2 evolution profile within a certain timeframe) do not drop within the first 7 hours, however, a slight deviation from the original activity (less than 10%) can be seen at longer reaction times.

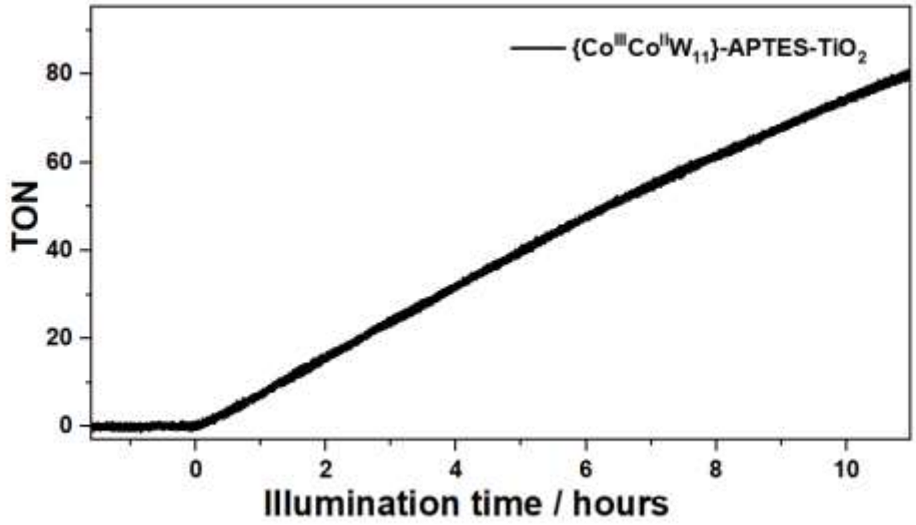

Figure S9: Long-term photocatalytic OER by {$Co^{III}Co^{II}W_{11}$}-APTES-TiO_2 under 365 nm illumination for more than 10 hours. Activity is expressed here as turnover numbers which correspond to number of O_2 molecules produced per POM molecule after the start of the illumination.

10. Post-catalytic characterization

Figure S10a features an exemplary ATR-FTIR spectrum of the {$Co^{III}Co^{II}W_{11}$}-APTES-TiO_2 composite after a WOC run. IR spectrum of the APTES-TiO_2 is subtracted for clarity to be able to focus on the POM-related bands. The spectrum of the composite after reaction (blue) features two strong bands in the region of 850-950 cm^{-1}. These correspond well to W-O-W and W=O vibrational modes characteristic of the {$Co^{III}Co^{II}W_{11}$} polyanion. Compared to the spectrum of the composite before reaction, however, one additional peak centered at 964 cm^{-1} can be seen, which we relate to either a blue-shifted W=O mode of the unattached {$Co^{III}Co^{II}W_{11}$} species (a possible peak split) or another component of the catalytic solution adsorbed on the sample surface after the catalytic run.

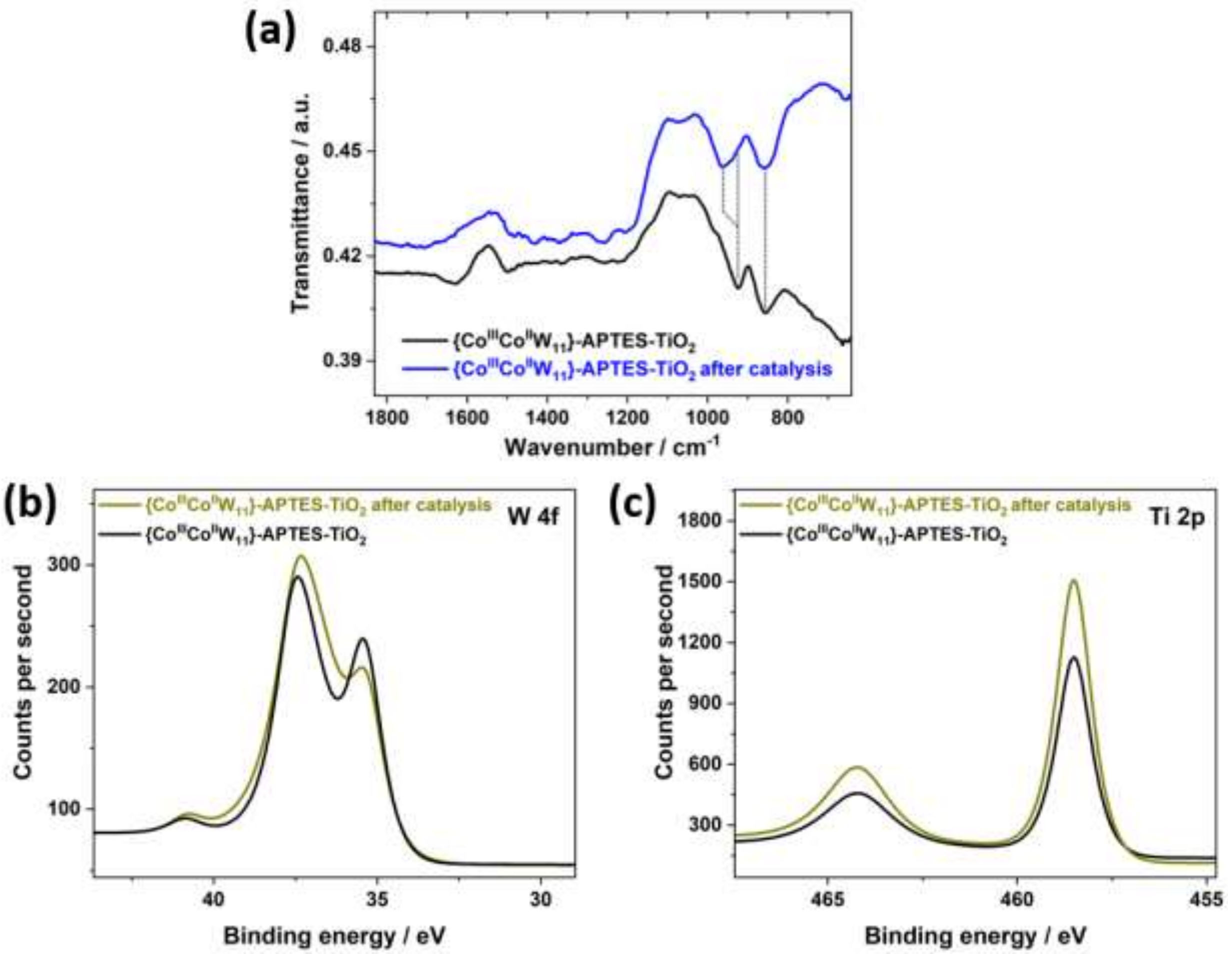

Figure S10: (a) ATR-FTIR spectra of the $\{Co^{III}Co^{II}W_{11}\}$-APTES-$TiO_2$ composites before (black) and after (blue) the photocatalytic WOC cycle, (b-c) XPS spectra of the $\{Co^{III}Co^{II}W_{11}\}$-APTES-$TiO_2$ composites before and after the photocatalytic WOC cycle showing W 4f (b) and Ti 2p (c) edges suitable for the identification of the composite components.

Figure S10b and c show as-recorded XPS profiles of the $\{Co^{III}Co^{II}W_{11}\}$-APTES-$TiO_2$ composites before and after WOC reaction featuring the most relevant W 4f and Ti 2p edges. The qualitative assessment confirms that both W and Ti edges have not been affected by the catalysis, which implies the presence of intact $\{Co^{III}Co^{II}W_{11}\}$-APTES-$TiO_2$ composite in the solid precipitate isolated after the WOC run. A more detailed look at the data based on the relative ratio of the W and Ti peaks (quantification data based on the peak areas), however, reveals a drop of W content by 24 % after WOC cycle, implying that a part (approx. quarter) of the POM clusters could have been detached from the APTES/TiO_2 surface over the course of the photocatalytic reaction.

In line with this observation, elemental analysis of the $\{Co^{III}Co^{II}W_{11}\}$-APTES-$TiO_2$ powder recovered after the WOC reaction using highly sensitive TXRF revealed 10.4±0.5 wt.% POM loading value (based on W amount), which is substantially lower compared to the 14.5±0.5 wt.% of $\{Co^{III}Co^{II}W_{11}\}$ that was present in the as-fabricated composite. The data are in line with XPS values and suggest that more than 25% of POM molecules have leached over the course of the reaction. Analysis of the liquid phase (reaction solution) further confirms the value.

Overall, these datasets strongly suggest that one out of four POM clusters have detached from APTES-TiO_2 surface over the course of the catalytic reaction. Considering the stable WOC rates of the $\{Co^{III}Co^{II}W_{11}\}$-APTES-$TiO_2$ composite upon hours-long illumination, these new findings suggest two different possibilities: (a) detached (leached) POM clusters continue to contribute to the WOC activity, which would imply that they are able to undergo charge-transfer interactions with TiO_2 nanoparticles in the suspension, in line with the results of the WOC experiments using a physical mixture of $\{Co^{III}Co^{II}W_{11}\}$ and TiO_2 (details in SI Section 9); (b) leaching of some of the POM clusters (possibly defined by the denticity of APTES attachment or particular density of the negatively charged POM species in the area) takes place during the initial stage of the photocatalytic run; the stable WOC performance observed in this work is thus related to the rest of the firmly attached POM clusters (~75% of the original loadings), while those clusters that leach do not contribute to WOC significantly. In addition to these possibilities, the leaching itself requires a more detailed investigation for the reason that causes the detachment: degradation of the ligand, displacement of N-Co bond, decomposition of the cluster into ions – can all be potential scenarios that call for a dedicated study.

11. PL investigation of the WOC mechanism

The WOC mechanism has been investigated using PL emission and time-resolved spectroscopy. In our system for PL measurements, $Na_2S_2O_8$ acts as an electron acceptor, while terephthalic acid (TA) – as OH radical (·OH) trap.

By extracting electrons (e^-) photogenerated in TiO_2, $Na_2S_2O_8$ helps to prevent the recombination of photogenerated e^- and holes (h^+), which allows the available h^+ to oxidize $^-OH/H_2O$ generating ·OH in the solution. Next, ·OH reacts rapidly with TA to form 2-hydroxyterephthalic acid (TA-OH), known for its strong PL in the visible range.

In many photocatalytic systems, ·OH is utilized for the degradation of organic compounds (i.e. pollutants, dyes). However, in this work – while the focus is given to OER – the formation of ·OH competes with the O_2 generation.

By utilizing PL emission spectroscopy (Figure 7), we have shown that in the case of $\{Co^{III}Co^{II}W_{11}\}$-APTES-$TiO_2$ composite – compared with bare TiO_2 – the photogenerated holes transfer to the attached POM clusters faster than they attack the surface-adsorbed ^-OH, which leads to a less effective ·OH formation, and hence more effective O_2 production. In contrast, bare TiO_2 generates a higher amount of ·OH during the reaction (Figure 7), which competes with water oxidation and inhibits O_2 formation. This tendency of TiO_2 to promote ·OH radical formation can be understood given that our experiments are performed at neutral pH.[19]

12. References

(1) Brunauer, S.; Emmett, P. H.; Teller, E. Adsorption of Gases in Multimolecular Layers. *J. Am. Chem. Soc.* **1938**, *60* (2), 309–319. https://doi.org/10.1021/ja01269a023.

(2) Song, F.; Ding, Y.; Ma, B.; Wang, C.; Wang, Q.; Du, X.; Fu, S.; Song, J. $K_7[Co^{III}Co^{II}(H_2O)W_{11}O_{39}]$: A Molecular Mixed-Valence Keggin Polyoxometalate Catalyst of High Stability and Efficiency for Visible Light-Driven Water Oxidation. *Energy Environ. Sci.* **2013**, *6* (4), 1170–1184. https://doi.org/10.1039/C3EE24433D.

(3) High Resolution XPS of Organic Polymers: The Scienta ESCA300 Database (Beamson, G.; Briggs, D.). *J. Chem. Educ.* **1993**, *70* (1), A25. https://doi.org/10.1021/ed070pA25.5.

(4) Wagner, C. D.; Naumkin, A. V.; Kraut-Vass, A.; Allison, J. W.; Powell, C. J.; Rumble Jr, J. R. NIST Standard Reference Database 20, Version 3.4 (Web Version). *Natl. Inst. Stand. Technol. Gaithersburg MD* **2003**, *20899*.

(5) Poblet, J. M.; López, X.; Bo, C. Ab Initio and DFT Modelling of Complex Materials: Towards the Understanding of Electronic and Magnetic Properties of Polyoxometalates. *Chem. Soc. Rev.* **2003**, *32* (5), 297–308. https://doi.org/10.1039/B109928K.

(6) Khan, M. I.; Tabussum, S.; Doedens, R. J.; Golub, V. O.; O'Connor, C. J. Functionalized Metal Oxide Clusters: Synthesis, Characterization, Crystal Structures, and Magnetic Properties of a Novel Series of Fully Reduced Heteropolyoxovanadium Cationic Clusters Decorated with Organic Ligands $[MV^{IV}_6O_6\{(OCH_2CH_2)_2N(CH_2CH_2OH)\}_6]X$ (M=Li, X=Cl·LiCl; M=Na, X=Cl·H_2O; M=Mg, X=2Br·H_2O; M=Mn, Fe, X=2Cl; M=Co, Ni, X=2Cl·H_2O). *Inorg. Chem.* **2004**, *43* (19), 5850–5859. https://doi.org/10.1021/ic049417m.

(7) Li, C.; Mizuno, N.; Yamaguchi, K.; Suzuki, K. Self-Assembly of Anionic Polyoxometalate–Organic Architectures Based on Lacunary Phosphomolybdates and Pyridyl Ligands. *J. Am. Chem. Soc.* **2019**, *141* (19), 7687–7692. https://doi.org/10.1021/jacs.9b02541.

(8) Li, L.; He, Y.; Zhang, Z.; Liu, Y. Nitrogen Isotope Fractionations among Gaseous and Aqueous NH^{4+}, NH_3, N_2, and Metal-Ammine Complexes: Theoretical Calculations and Applications. *Geochim. Cosmochim. Acta* **2021**, *295*, 80–97. https://doi.org/10.1016/j.gca.2020.12.010.

(9) Patel, A.; Sadasivan, R.; Patel, J. Chiral Phosphotungstate Functionalized with (S)-1-Phenylethylamine: Synthesis, Characterization, and Asymmetric Epoxidation of Styrene. *Inorg. Chem.* **2021**, *60* (15), 10979–10989. https://doi.org/10.1021/acs.inorgchem.1c00636.

(10) Wang, W.; Qiu, Y.; Xu, L. Supramolecular Coexistence of Co(II) and Ag(I) Complexes Based on Polyoxotungstate and Imidazoles: Synthesis, Crystal Structure, and Spectroscopic Study. *J. Coord. Chem.* **2014**, *67* (5), 797–806. https://doi.org/10.1080/00958972.2014.908464.

(11) Miao, H.; Dong, Y.; Chen, Z.; He, X.; Hu, G.; Xu, Y. Design, Synthesis and Excellent Third-Order NLO Properties of Two New Polyoxometalates Constructed from Keggin Polyanions Bonded by a Solvent Molecule. *Dalton Trans.* **2016**, *45* (32), 12717–12722. https://doi.org/10.1039/C6DT02320G.

(12) Liu, H.; Gómez-García, C. J.; Peng, J.; Sha, J.; Wang, L.; Yan, Y. A Co-Monosubstituted Keggin Polyoxometalate with an Antenna Ligand and Three Cobalt(II) Chains as Counterion. *Inorganica Chim. Acta* **2009**, *362* (6), 1957–1962. https://doi.org/10.1016/j.ica.2008.09.014.

(13) Si, C.; Liu, F.; Yan, X.; Xu, J.; Niu, G.; Han, Q. Designing a Polyoxometalate-Incorporated Metal–Organic Framework for Reduction of Nitroarenes to Anilines by Sequential Proton-Coupled Electron Transfers. *Inorg. Chem.* **2022**, *61* (13), 5335–5342. https://doi.org/10.1021/acs.inorgchem.2c00106.

(14) Breibeck, J.; Bijelic, A.; Rompel, A. Transition Metal-Substituted Keggin Polyoxotungstates Enabling Covalent Attachment to Proteinase K upon Co-Crystallization. *Chem. Commun.* **2019**, *55* (77), 11519–11522. https://doi.org/10.1039/C9CC05818D.

(15) Hu, J.-J.; Wang, L.; Chen, B.-N.; Chi, G.-X.; Zhao, M.-J.; Li, Y. Transition Metal Substituted Polyoxometalates as α-Glucosidase Inhibitors. *Eur. J. Inorg. Chem.* **2019**, *2019* (28), 3270–3276. https://doi.org/10.1002/ejic.201900306.

(16) Baker, L. C. W.; Figgis, J. S. New Fundamental Type of Inorganic Complex: Hybrid between Heteropoly and Conventional Coordination Complexes. Possibilities for Geometrical Isomerisms in 11-, 12-, 17-, and 18-Heteropoly Derivatives. *J. Am. Chem. Soc.* **1970**, *92* (12), 3794–3797. https://doi.org/10.1021/ja00715a047.
(17) Johnson, B. J. S.; Stein, A. Surface Modification of Mesoporous, Macroporous, and Amorphous Silica with Catalytically Active Polyoxometalate Clusters. *Inorg. Chem.* **2001**, *40* (4), 801–808. https://doi.org/10.1021/ic991440y.
(18) Vilarrasa-García, E.; Cecilia, J. A.; Moya, E. M. O.; Cavalcante, C. L.; Azevedo, D. C. S.; Rodríguez-Castellón, E. “Low Cost” Pore Expanded SBA-15 Functionalized with Amine Groups Applied to CO_2 Adsorption. *Materials* **2015**, *8* (5), 2495–2513. https://doi.org/10.3390/ma8052495.
(19) Zhang, J.; Nosaka, Y. Photocatalytic Oxidation Mechanism of Methanol and the Other Reactants in Irradiated TiO_2 Aqueous Suspension Investigated by OH Radical Detection. *Appl. Catal. B Environ.* **2015**, *166–167*, 32–36. https://doi.org/10.1016/j.apcatb.2014.11.006.
